# Herbal Medicines for Parkinson's Disease: A Systematic Review of Randomized Controlled Trials

**DOI:** 10.1371/journal.pone.0035695

**Published:** 2012-05-15

**Authors:** Tae-Hun Kim, Ki-Ho Cho, Woo-Sang Jung, Myeong Soo Lee

**Affiliations:** 1 Department of Cardiovascular & Neurologic Diseases, College of Oriental Medicine, Kyung Hee University, Seoul, South Korea; 2 Korea Institute of Oriental Medicine, Daejeon, South Korea; National Institutes of Health, United States of America

## Abstract

**Objective:**

We conducted systematic review to evaluate current evidence of herbal medicines (HMs) for Parkinson's disease (PD).

**Methods:**

Along with hand searches, relevant literatures were located from the electronic databases including CENTRAL, MEDLINE, EMBASE, CINAHL, AMED, PsycInfo, CNKI, 7 Korean Medical Databases and J-East until August, 2010 without language and publication status. Randomized controlled trials (RCTs), quasi-randomized controlled trials and randomized crossover trials, which evaluate HMs for idiopathic PD were selected for this review. Two independent authors extracted data from the relevant literatures and any disagreement was solved by discussion.

**Results:**

From the 3432 of relevant literatures, 64 were included. We failed to suggest overall estimates of treatment effects on PD because of the wide heterogeneity of used herbal recipes and study designs in the included studies. When compared with placebo, specific effects were not observed in favor of HMs definitely. Direct comparison with conventional drugs suggested that there was no evidence of better effect for HMs. Many studies compared combination therapy with single active drugs and combination therapy showed significant improvement in PD related outcomes and decrease in the dose of anti-Parkinson's drugs with low adverse events rate.

**Conclusion:**

Currently, there is no conclusive evidence about the effectiveness and efficacy of HMs on PD. For establishing clinical evidence of HMs on PD, rigorous RCTs with sufficient statistical power should be promoted in future.

## Introduction

Parkinson's disease (PD) is an age-related neurodegenerative disorder that pathological feature is basically related on the progressive degradation of dopamine production in substantia nigra. The clinical manifestation includes bradykinesia (especially having difficulties in initiating movement), hypokinesia (lose of facial expression), rigidity, rest tremor (pill-rolling movement of the forearm) and non-motor features including depression, psychosis autonomic dysfunction [1].

Because of diversity of the studies with different population and methodology, the prevalence rate and incidence varies broadly in difference studies. In the European countries, crude prevalence rate estimates ranged from 65.6 per 100,000 to 12,500 per 100,000 and annual incidence estimates ranged from 5 per 100,000 to 26 per 100,000 [2]. On the contrary, prevalence rate and incidence rate is slightly lower in Asian countries. It was reported that a standardized all-age prevalence of was 51.3 to 176.9 per 100,000 and the standardized incidence rates were 8.7 per 100,000 person-years [3]. The prevalence increases progressively along with the age. According to the cross-sectional study of United Kingdom in 2000, the prevalence was only 20/100,000 in below 50 years old, but 342/100,000 in sixties and 1265/100,000 in over eighties [4]. Considering that medical expenditure for PD is one of the highest ranked neurologic diseases, PD might be a serious socioeconomic burden in future aging society [5]–[7].

Herbal products for PD have been used worldwide in traditional medicine [8]. Especially in China, records of herbal prescriptions for treating PD might date back to about 2200 years ago [9] and current literatures reflect active use of HM for PD until now [10], [11]. Practitioners and patients employ HM as an adjuvant therapy of conventional treatment for the purpose of reducing dose of dopaminergic drugs, adverse event related to prolonged usage of dopaminergic agents and improving PD symptoms [10].

Previous literature reviews have been evaluated the clinical evidence of HM including traditional Chinese medicine (TCM) [9], [10], [12], [13]. However these reviews did not adopt explicit search strategy so all the possible studies might not be located. Information on the herbal remedies was not presented in details. In addition, any results on the effect size were not reported. From these limitations, these reviews failed to suggest the global picture of HMs for treating PD [9], [10], [12], [13].

The purpose of this review is to evaluate the current evidence level thorough explicit systematic review method and to present necessary information to outline the benefit and harmfulness of HMs for PD patients.

## Methods

### Types of studies

Randomized controlled trials (RCTs), quasi-randomized controlled trials and randomized crossover trials, which evaluate HMs for idiopathic PD were included in this review.

### Types of participants

Participants of any age and sex with idiopathic PD were considered to be included. Diagnosis of PD is necessary to be done considering clinical symptoms and radiological examinations through the standard diagnostic criteria such as UK Parkinson's Disease Brain Bank criteria [14] or the Diagnosis and Differential Diagnosis of Parkinson's Disease and Parkinsonism from the Chinese Colloquium of Extracorticospinal Tract Diseases [15].

### Types of intervention

Studies which used HMs as the only intervention or combination therapy with conventional medicine were included. In this review, HMs was defined as products originated from botanical sources such as whole plants or their adjuncts: seed, root, flower, bud and leaf [16]. In the boundary of HM, low or manufactured single or complex medicinal plants, plants extracts and plant preparations for the purpose of treatment were included regardless of drug preparation: extracts, decoction, tablet, capsule, pill, powder, injection and etc [17]. However, drugs, which were consisted of synthesized compounds, were excluded. Complex intervention with acupuncture, moxibustion and other CAM treatments was excluded. There is no restriction on dosage including frequency, dose, intensity and duration. Comparator interventions were conventional drugs or treatment, surgery, placebo, usual care or no treatment. Comparison between different types of HMs was excluded. Conventional drugs include levodopa (both of the immediate-release and modified-release), dopamine agonists, monoamine oxidase B inhibitors, catecho-O methyltransferase inhibitor (COMT) and amatadine etc.

### Types of outcome measures

Following primary and secondary outcomes were pre-identified at the protocol stage:

#### Primary outcomes

Total Unified Parkinson's Disease Rating Scale score (Total UPDRS score)

Global improvement of symptoms

#### Secondary outcomes

UPDRS I, II, III and IV sub-scores

Webster scale

Hoehn and Yahr (H&Y) staging

Non-movement problems: total non-motor symptoms score value (NMSQuest), frequency of Nausea & vomiting or constipation

Mean levodopa or bromocriptine usage

Depression scales: Hamilton depression rating scale (HAM-D)

Quality of life measures: Parkinson's Disease Questionnaire–39 (PDQ-39) and EuroQol (EQ-5D)

Adverse events rate

### Search methods for identification of studies

The following databases were searched until August, 2010. Any language, document format and date restrictions didn't included in the search strategy to minimize possible bias: Cochrane Central Register of Controlled Trials (CENTRAL), MEDLINE, EMBASE, CINAHL (Cumulative Index of Nursing and Allied Health Medicine), AMED (Allied and Complementary Medicine Database), PsycInfo, China Academic Journal in China National Knowledge Infrastructure (CNKI), China Doctor/Master's Dissertation Database in CNKI, China Proceedings of Conference Database in CNKI, Korean Medical Databases (Korean Studies Information, DBPIA, Korea Institute of Science and Technology Information, Research Information Centre for Health Database, KoreaMed and the Korean National Assembly Library, Korean traditional knowledge portal) and J-East. Related gray literature and references of included studies were hand searched. Searching strategy for medline was as follows:

### Medline (Pubmed) search strategy

#1 Parkinson Disease [mh]

#2 Parkinson$ [tiab]

#3 or/1-2

#4 Medicine, African Traditional [mh]

#5 Medicine, Arabic [mh]

#6 Medicine, Ayurvedic [mh]

#7 Medicine, Kampo [mh]

#8 Medicine, Korean Traditional [mh]

#9 Medicine, Tibetan Traditional [mh]

#10 Medicine, Mongolian Traditional [mh]

#11 Herbal Medicine [mh]

#12 Phytotherapy [mh]

#13 Drugs, Chinese Herbal [mh]

#14 Plants, Medicinal [mh]

#15 Plant Extracts [mh]

#16 Ethnobotany [mh]

#17 Ethnopharmacology [mh]

#18 Plants [mh]

#19 herb$ [tiab]

#20 (Plant Leave$ or Flower$ or Plant Root$ or Fruit$ or Seed$) [tiab]

#21 or/4-20

#22 3 and 21

#23 randomized controlled trial [pt]

#24 controlled clinical trial [pt]

#25 randomized [tiab]

#26 placebo [tiab]

#27 drug therapy [sh]

#28 randomly [tiab]

#29 groups [tiab]

#30 or/23-29

#31 animals [mh] not (humans [mh] and animals [mh])

#32 30 not 31

### Data extraction, risk of bias assessment and analysis

From the included studies, data describing disease duration and severity, interventions for the treatment and control groups, summary of the outcomes, last follow up period and recipes of HMs were extracted according to the pre-defined data extraction form by two independent authors (THK and WSJ). Any disagreement between two authors was solved by discussion. Risk of bias was assessed in 6 domains: random sequence generation, allocation concealment, blinding of participants, incomplete outcome data, selective reporting and other bias. Two independent authors (THK and KHC) assessed the risk of bias according to the criteria of Cochrane collaboration [18]. Any conflict was discussed and there was no disagreement between two authors.

Estimated effect size for each outcome of included HM was calculated comparing with each control intervention individually. Individual data were combined for meta-analysis if the studies adopted the same HM and control intervention. Dichotomous data were presented as relative risk (RR) and continuous outcomes as mean difference (MD) with 95% CI. Analysis was conducted with Review manager 5.1 (Copenhagen: The Nordic Cochrane Centre, The Cochrane Collaboration, 2011).

## Results

### Description of included studies

**Figure 1:**
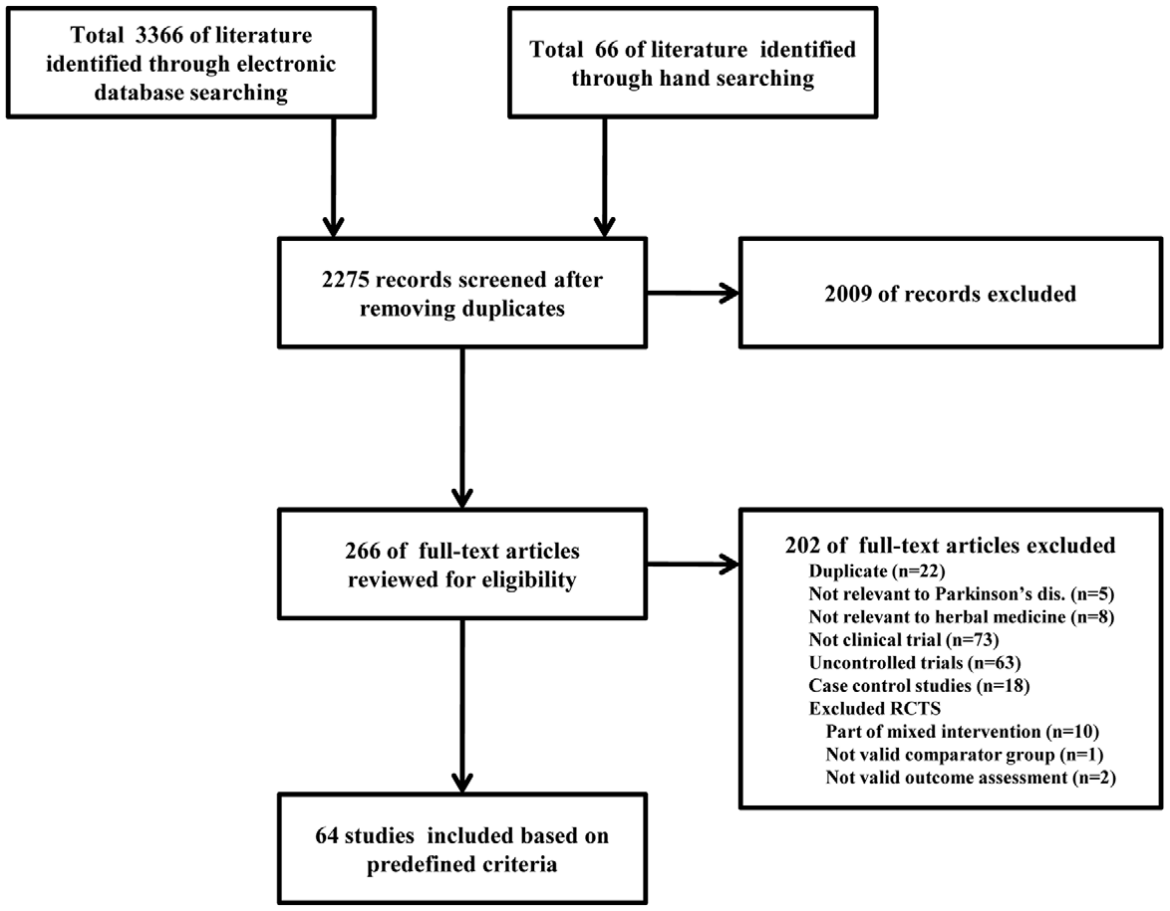
Flow chart of information for this systematic review.

Total 3432 of relevant literature were identified from electronic databases and hand searching. Among them, 64 studies (total 4024 PD patients) were included based on the predefined criteria (figure 1).

All the included studies were classified into one of four types according to the trial design: HM versus placebo [19], [34], HM versus conventional medicine [30], [43], [44], [53], [57], [71], [73], [77], HM plus active drug versus active drug [20]–[23], [25]–[27], [29], [31]–[33], [35]–[38], [41], [42], [45], [48]–[50], [52], [54], [55], [56], [58]–[62], [64]–[69], [72], [74]–[76], [79]–[82] or HM plus active drug versus placebo plus active drug [11], [24], [28], [34], [39], [40], [46], [47], [51], [63], [70], [78]. Among the 64 studies, one was conducted in Ecuador [19], another was in UK [24] and other 62 were in China. All the included studies were RCTs except two quasi-RCTs [31], [54] and one cross-over study [24].

### Interventions

A total 59 kinds of herbal preparations were identified in the included studies (table S1). Among them, several different herbal recipes which were prescribed individually according to each patient were used in three studies [80]–[82]. All the identified interventions were complex medications which were composed of three to twenty herbs. Only in two studies, single herbal extracts were tested [19], [24]. In majority of included studies, treatment was provided during 3 or 6 months (ranging 1 month to 1 year). One study assessed the short term effect of herbal extract, tested shortly after taking medication once [19]. Control interventions were mainly conventional treatment with anti-Parkinson drugs or Placebo [11], [19], [24], [28], [34], [39], [40], [46], [47], [51], [63], [70], [78]. In 58 trials, several anti-Parkinson drugs were provided to PD patients, combined with HMs and 8 studies tested single HMs without conventional treatment (table S2) [30], [43], [44], [53], [57], [71], [73], [77].

### Risk of bias

There is no study with row risk of bias in all the 6 domains and no study showed low risk of biases in 5 domains. There were 14 studies which showed low risk of bias in the domain of random sequence generation [21], [24], [28], [34], [46]–[50], [55], [70], [74], [78], [83]. Allocation concealment was conducted adequately in 5 studies [21], [24], [50], [55], [78]. 13 studies described about blinding issues [19], [24], [28], [34], [39], [40], [46], [47], [51], [63], [68], [70], [78]. In 10 studies [24], [25], [47], [53]–[55], [65], [70], [75], [83], incomplete outcome data such as numbers of participants who were included, finished treatment and were dropped out were well described. The selective reporting domain was not evaluated clearly because we could not locate any registered protocol for all the included studies. Other risk of bias such as baseline imbalances were properly described in the included studies except in 12 studies [19], [22], [24], [31], [36], [41], [47], [49], [59], [66], [72], [76].

In addition to this, several details were also evaluated to describe the methodological quality of the included studies. No study did statistical analysis on intention-to-treat basis. In only one study, sample size calculation was conducted for enough statistical power [24]. 8 studies referred to the ethical issues such as prior review by institutional review board [19], [24] or written informed consent from participants [19], [21], [24], [34], [55], [65], [83].

### Effects of interventions 1: HM versus placebo [19], [34]

**Figure 2:**
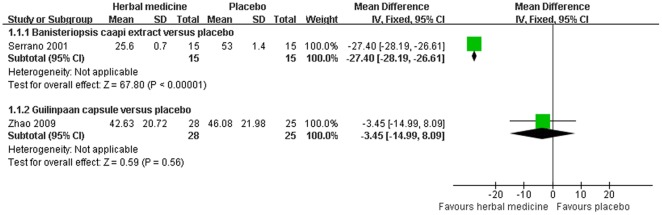
Herbal medicine versus placebo, total UPDRS score.

2 HMs were compared with placebo in 2 studies [19], [34]. Global improvement of symptoms and total UPDRS score were assessed for the efficacy of HMs in 2 studies (figure 2) [19], [34]. Banisteriopsis caapi extract showed significant improvement in total UPDRS score 4 hrs after the one time treatment (MD −27.40, [−28.19, −26.61]) [19]. 3 months of Guilingpaan capsule treatment did not show statistically significant difference in global symptoms (RR 1.30, [0.93, 2.06]) and total UPDRS score (MD −3.45, [−14.99, 8.09]), compared with placebo control [34].

### Effects of interventions 2: HM versus conventional medicine [30], [43], [44], [53], [57], [71], [73], [77]

8 different HMs were tested compared with conventional drug therapy [30], [43], [44], [53], [57], [71], [73], [77]. Global improvement of symptoms, Webster scale and total UPDRS score were evaluated in these studies. Among them, herbal formula Qingxinhuatan tang only showed statistically significant improvement in global symptoms (RR 1.33, 95% CI [1.04, 1.72]) compared with madopar therapy [53]. Herbal formula Kangzhenzhijing capsule [43], Kanli tang [44] and Ziyinxifeng granule [77] reported significant improvement in Webster score (MD −1.80, [−3.40, −0.20], −2.40, [−4.29, −0.51] and −0.50, [−0.90, −0.10], respectively). Herbal formula Zhichanshudu tang showed significant improvement in total UPDRS score (MD −9.16, [−10.68, −7.64]) [73].

### Effects of interventions 3: HM plus active drug versus active drug [20]–[23], [25]–[27], [29], [31]–[33], [35]–[38], [41], [42], [45], [48]–[50], [52], [54], [55], [56], [58]–[62], [64]–[69], [72], [74]–[76], [79]–[82]

In 44 studies, HM combined with conventional drug therapy was compared with conventional drug treatment [20]–[23], [25]–[27], [29], [31]–[33], [35]–[38], [41], [42], [45], [48]–[50], [52], [54], [55], [56], [58]–[62], [64]–[69], [72], [74]–[76], [79]–[82]. Herbal formula Guilingpaan wan was evaluated in 3 studies [35]–[37], Xifengdingzhan wan in 3 studies [61], [63], [84] and other HMs was used once:

Three months of Guilingpaan wan medication with madopar showed significant improvement in global symptoms (RR 1.45, [1.20, 1.77]) [35]–[37] and total UPDRS score (MD −9.97, [−16.68, −3.25] [35], [36] compared with madopar alone.

Xifengdingzhan wan with madopar was administered for 3 months [61], [84] and for 1 month [63], respectively. Xifengdingzhan wan combination therapy improved significantly in global symptoms (RR 1.47, [1.20, 1.81]) [61], [63] and total UPDRS score (MD −5.41, [−6.96, −3.86]) [61], [84].

#### Global improvement of symptoms

33 studies assessed global improvement of symptoms. Among them, 3 studies [80]–[82] compared the effectiveness of combination treatment on global symptoms with individualized herbal recipes plus conventional drug therapy versus single drug therapy, and significant improvement was observed in 1 study only [79] (RR 2.88, [1.90, 4.34]). The other studies tested different HMs with conventional drug therapies and 10 herbal recipes including those described earlier, showed statistically significant improvement in global symptoms [33], [35]–[37], [41], [50], [52], [55], [60], [61], [63], [65], [79]: Fufangjangzhan wan plus madopar (RR 1.40, [1.16, 1.70]) [33], Kanapa granule with madopar (RR 3.13, [1.79, 5.46]) [41], Pabing formula 2 plus madopar (RR 2.80, [1.15, 6.80]) [50], Peibuganshen recipe plus madopar and artane (RR 1.89, [1.01, 3.55]) [52], Shudipingzhan tang plus Xiewu capsule plus levodopa (RR 2.27, [1.60, 3.23]) [55], Xifengdinzhan tang with madopar (RR 1.32, [1.01, 1.72]) [60] and Xifengzhizhan tang plus madopar (RR 1.50, [1.08, 2.08]) [65].

#### Total UPDRS score

**Figure 3:**
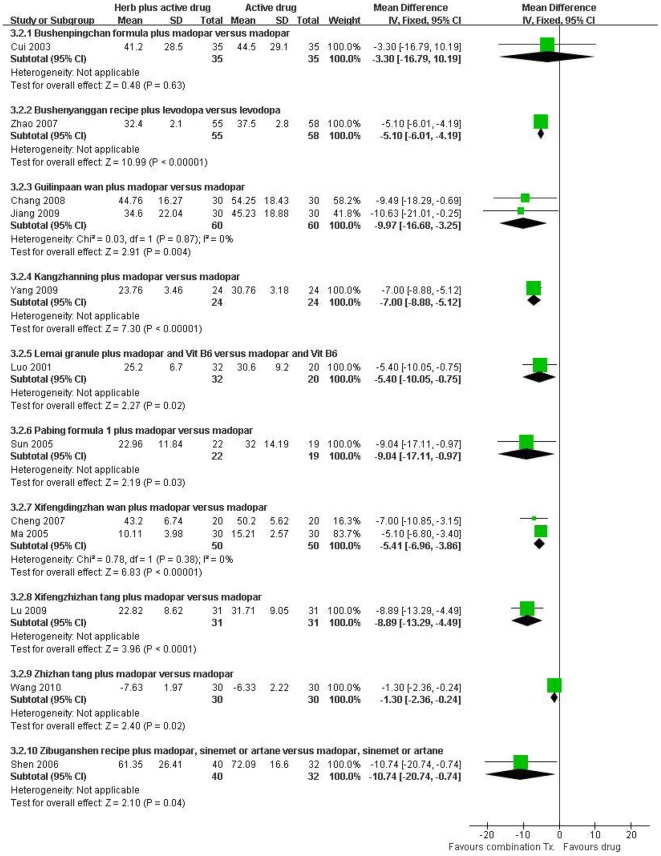
Herbal medicine plus active drug versus active drug, total UPDRS score.

In total 12 studies [20], [21], [35], [36], [42], [45], [48], [61], [65], [74], [75], [84], 10 HM plus conventional drug therapies were compared with single drug therapy for the total UPDRS score and among them, 9 HMs including Guilingpaan wan and Xifengdingzhan wan showed significant improvement in the score: Bushenyanggan recipe plus levodopa (MD −5.10, [−6.01, −4.19]) [21], Kangzhanning plus madopar (MD −7.00, [−8.88, −5.12]) [42], Lemai granule plus madopar and Vit B6 (MD −5.40, [−10.05, −0.75]) [45], Pabing formula 1 plus madopar (MD −9.04, [−17.11, −0.97]) [48], Xifengzhizhan tang plus madopar (MD −8.89, [−13.29, −4.49]), Zhizhan tang plus madopar (MD −1.30, [−2.36, −0.24]) [74] and Zibuganshen recipe plus madopar, sinemet or artane (MD −10.74, [−20.74, −0.74], figure 3) [75].

#### Webster scale

Webster scale was assessed in 4 studies [26], [52], [66], [75] and combination therapy showed better effect than conventional therapy alone in 3 studies: Peibuganshen recipe plus madopar and artane (MD −3.10, [−5.00, −1.20]) [52], Yangganxifeng recipe plus madopar (MD −3.00, [−4.14, −1.86]) [66] and Zibuganshen recipe plus madopar, sinemet or artane (MD −2.86, [−5.68, −0.04]) [75].

#### Hoehn & Yahr score

Hoehn & Yahr score was assessed in 2 studies [21], [38]. However there was no significant improvement in these studies.

#### The dose of anti-Parkinson drug

**Figure 4:**
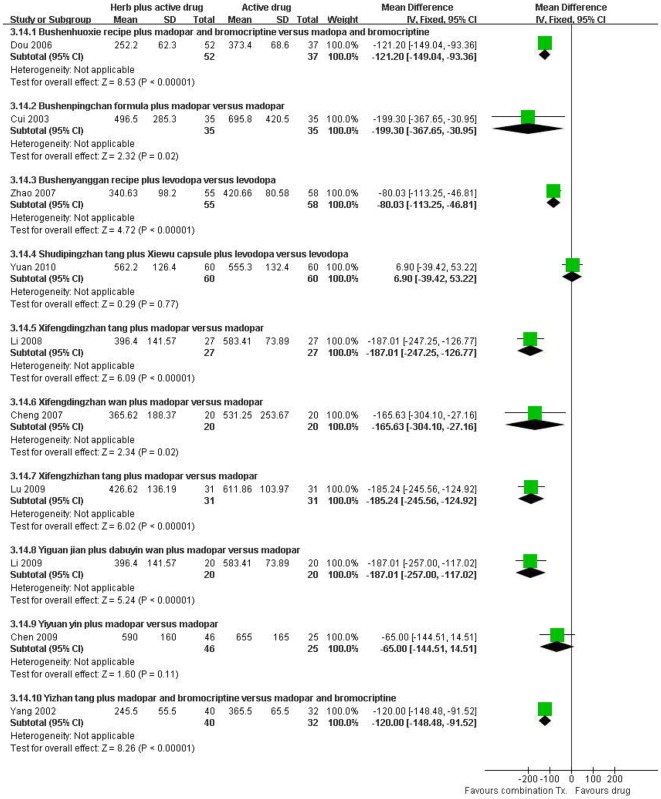
Herbal medicine plus active drug versus active drug, mean dose of levodopa (madopar).

The dose of anti-Parkinson drug including levodopa (madopar) or bromocriptine was compared between combination treatment and conventional drug therapy groups (figure 4). Mean dose of levodopa (madopar) was assessed in 10 studies [20], [21], [55], [60], [65], [67]–[69], [83], [84] and combination therapies decreased the dose significantly in 8 studies: Bushenpingchan formula (MD −199.30, [−367.65, −30.95]) [20], Bushenhuoxie recipe (MD −121.20, [−149.04, −93.36]) [83], Bushenyanggan recipe (MD −80.03, [−113.25, −46.81]) [21], Xifengdingzhan tang (MD −187.01, [−247.25, −126.77]) [60], Xifengdingzhan wan (MD −165.63, [−304.10, −27.16]) [84], Xifengzhizhan tang (MD −185.24, [−245.56, −124.92]) [65], Yiguan jian plus dabuyin wan (MD −187.01, [−257.00, −117.02]) [67] and Yizhan tang (MD −120.00, [−148.48, −91.52]) [69].

#### Non-movement problems

Non-movement problems such as nausea and constipation were evaluated in several studies. 1 study adopted a validated systemic tool i.e. Parkinson's disease non-movement questionnaire (NMSQuest) for non-movement symptoms: Yiyuan yin plus madopar was compared with single madopar therapy. However, there was no significant difference between 2 groups in NMSQest value (MD −1.00, [−3.19, 1.19]) [68]. 4 HMs plus conventional drug therapies were tested for the effect of reducing nausea and vomiting [35]–[37], [55], [75], [83]. Among them, 2 HMs showed favorable results: Bushenhuoxie recipe plus madopar and bromocriptine (RR 0.40, [0.20, 0.81]) [83], Zibuganshen recipe plus madopar and sinemet or artane (RR 0.36, [0.14, 0.94]) [75].

7 herbal remedies with anti-Parkinson drugs were evaluated on constipation in PD patients [29], [36], [42], [55], [68], [75], [83] and 3 recipes showed better results: Bushenhuoxie recipe plus madopar and bromocriptine (RR 0.11, [0.03, 0.46]) [83], Dingzhen tang_2 plus conventional treatment (RR 0.48, [0.29, 0.78]) [29] and Kangzhanning plus madopar (RR 0.50, [0.28, 0.88]) [42].

#### Anti-depressive effect

Anti-depressive effect of combination therapy in PD patients was tested in 1 study. Compared with paroxetine treatment alone, Chaihushugan san plus paroxetine showed significant improvement in HAM-D score (MD −4.10, [−5.17, −3.03]) after 8 weeks of treatment with low adverse event rate (RR 0.48, [0.31, 0.74]) [25].

#### Total adverse event rate

Total adverse event rate was assessed in 8 studies [25], [31], [38], [45], [54], [56], [61], [84] and 5 studies (4 HMs) reported significantly low rate of adverse event: Chaihushugan san plus paroxetine (RR 0.48, [0.31, 0.74]) [25], Dingzhen tang_4 plus madopar and artane (RR 0.39, [0.18, 0.83]) [31], Ruogantongluo tang plus madopar (RR 0.36, [0.14, 0.89]) [45] and Xifengdingzhan wan plus madopar (RR 0.41, [0.21, 0.80]) [61], [84].

### Effects of interventions 4: HM plus active drug versus placebo plus active drug [11], [24], [28], [34], [39], [40], [46], [47], [51], [63], [70], [78]

12 studies compared the effect of HM plus active drug and those of placebo plus active drug [11], [24], [28], [34], [39], [40], [46], [47], [51], [63], [70], [78]. In 8 studies, global improvement of symptoms was compared between HMs and placebo controls both of which were combined with active drugs [28], [34], [39], [46], [47], [51], [63], [78]. Among them, significant improvement was observed in 5 studies: Jiaweidadingfeng zhu plus madopar (RR 3.83, [1.82, 8.05]) [39], Naokangning capsule plus madopar (RR 1.53, [1.02, 2.31]) [46], Nuzhenyangyin granule plus madopar and artane (RR 1.53, [1.09, 2.16]) [47], Pabing formula 3 plus madopar (RR 2.60, [1.06, 6.39]) [51] and Xifengdingzhan wan plus madopar (RR 1.44, [1.11, 1.87]) [63].

Total UPDRS score was evaluated in 5 studies [29], [46], [63], [70], [78] and 2 HMs showed significant improvement comparing with placebo plus active drug treatment: Naokangning capsule plus madopar (MD −6.00, [−11.24, −0.76]) [46] and Xifengdingzhan wan plus madopar (MD −5.10, [−6.57, −3.63]) [63].

SF-36 questionnaires were assessed for evaluating quality of life for PD patients in 1 study [46]. Compared with placebo plus active treatment, Naokangning capsule plus madopar improved significantly in the subsections for the physical functioning (58.74 (14.16) in Naokangning plus madopar group, 46.83 (16.43) in placebo plus madopar, P<0.05), social functioning (77.26 (27.57), 64.63 (20.39), P<0.05), role limitations due to physical health (68.59 (16.33), 49.62 (18.25), P<0.05) and vitality (67.21 (18.05), 48.92 (23.49), P<0.05) [46].

## Discussion

Among the included 64 RCTs, 59 herbal preparations were used for treating PD. Majority of studies were conducted in China and HMs were composed of multiple herbs in almost all studies. Only 5 HMs were used several times but other studies were conducted with high heterogeneities in herbal preparations and study design. HMs was used with conventional drugs concomitantly in majority of the included studies. Efficacy of HMs was tested in 2 studies by comparing with placebo control. Banisteriopsis caapi extract showed short-term effect in improving UPDRS score but 3 months of Guilingpaan capsule medication did not have statistically significant differences in PD symptoms [19], [34]. HMs were directly compared with conventional drug therapy in 8 studies and only Qingxinhuatan tang improved general symptoms of PD significantly in one study [53]. Combination therapy with HMs and anti-Parkinson drugs showed significant improvement in the assessment of general symptoms and UPDRS scores and decrease in the dose of anti-Parkinson drugs generally. Adverse events rate also decreased significantly in combination therapies.

**Figure 5:**
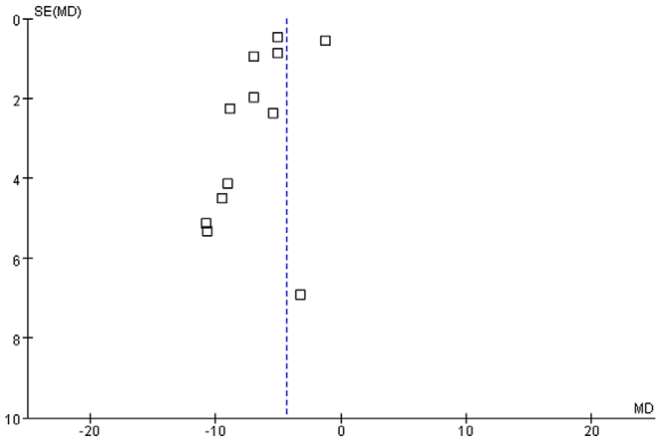
Herbal medicine plus active drug versus active drug, funnel plot for total UPDRS score.

**Figure 6:**
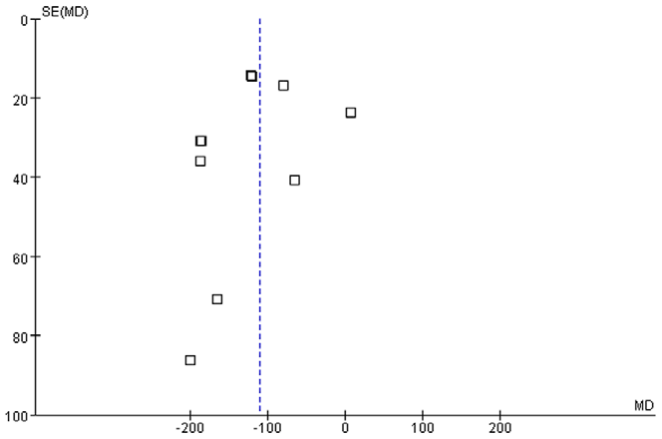
Herbal medicine plus active drug versus active drug, funnel plot for mean dose of levodopa (madopar).

Compared with the previous systematic review in 2006 [85], the number of conducted RCTs increased steeply from 9 to 64. However, as with before, the result of this systematic review cannot suggest conclusive evidence of HMs because of several limitations the included studies had. Firstly, only a small number of preparations were used twice or more and most of the HMs were tested only once. A huge heterogeneity in the herbal composition, drug formulation, dose, duration, combined therapy and control interventions was observed between the studies, which were the main obstacles to estimate the general effect size of herbal remedies. Secondly, significant improvement in the methodological qualities of included studies was not observed compared with the previous review. As pointed out before, most of the studies did not report about allocation concealment process. Incomplete outcome data including drop out were not reported in 53 studies. Because any pre-registered protocols of included trials were not located, selective outcome reporting could not be judged clearly in all the studies. Only one studies adopted ITT analysis for statistics. In this review, except 2 studies, all the searched studies were conducted in China. There have been debates about the results of clinical trials in some countries including China, which may be originated from their methodological flaws and publication bias [86]. Trials with less methodological rigor prone to exaggerate treatment effect than rigorous studies [87]. Thirdly all the included studies tested only small sample size without proper power calculation. Generally studies with small sample may not detect clinically important difference between interventions [88]. Among the included studies in this review, some HMs did not show statistically significant effect in favor of herbal preparations compared with placebo or conventional treatment which may be originated from their insufficient sample size. Fourthly there may be a possibility of publication bias in this review. We checked funnel plots of two key outcomes i.e. total UPDRS scores and mean dose of levodopa in the comparison of HM plus active drug and active drug only. Both of the funnel plots appeared asymmetric, which might be originated from publication bias or poor methodological qualities of the included studies (figure 5 and 6).

In these points, the findings of this systematic review should be interpreted carefully.

Even with several limitations, this review also has strength as well. We did comprehensive search regardless of language and publication types to locate every possible RCT. We failed to suggest overall estimates of treatment effects on PD but herbal recipes and estimates of effect measures on the separate outcomes for individual HMs were presented to provide information in detail, which may be available to suggest a basic source for judging the possibility of treating PD and after all may offer a principle data to find out new drug candidates for PD [89].

There are several issues on the HMs for the treatment of PD, which should be discussed more. We found that herbal recipes were generally used as adjuvant treatments to conventional drug therapy which may contribute to improve the PD symptoms and to decrease dose of anti-Parkinson drugs and adverse events rate. Many PD patients experience dyskinesia and symptom fluctuation from the long-term treatment with levodopa [90]. Combination therapy with some HMs showed significant or marginal effect in reducing complications of levodopa (UPDRS IV) [41]. The dose of levodopa is also one of the important clinical considerations because PD needs lifelong treatment generally. Considering wearing-off symptoms, it would be helpful for both patients and clinicians to reduce dose of levodopa for managing PD symptoms [1]. In this point, HM may be considered as a good adjunctive treatment to conventional drugs for PD. On the contrary, HMs for treating PD needs to equip standardized criteria for use to ensure the good quality of the products. It includes the problems related to the original species of individual herbs, chemical compositions and indexes, preparations and indications for herbal products. Chinese formulas were composed of many herbs and there were a wide heterogeneity in the HMs between the studies included in this review. In this situation, standardization of natural products used in the clinical trials is one of the most necessary factors for demonstrating good reproducibility of the research result in real clinical practices. Pharmacological researches including in vivo and in vitro studies should be promoted to support biological mechanism of herbal products as well. Adverse effects (AE) relevant to herbal recipes also need to be described clearly. AEs of the drugs for PD are well documented and are frequently reported of high occurrence rate in conventional medicine [1]. For example, in one study, dyskinesia was experienced in up to 40% of PD patients after 4 to 6 years of levodopa treatment [90]. In this review, occurrence of adverse events was relatively low in the combination therapies. This result may be originated from the fact that observation periods of the included studies were relatively short. Safety issues need to be one of the primary outcomes and clearly be described in future trials with long term observational period.

Future clinical trials of HMs on PD need to improve methodological quality to evaluate clinical benefit and harm properly. Random sequence generation and double blinding should be ensured in study design and results need to be reported well according to the CONSORT statement [91]. Protocol registration before start of clinical trials is also important to guarantee the transparency of the study finding through avoiding selective outcome reporting and publication bias [92]. Along with trials for efficacy in which single HM is compared with placebo control, comparative effectiveness studies using combination therapy with conventional drug therapies also need to be conducted to reflect clinical situation as it is.

## Supporting Information

Table S1 The preparation of the herbal medicines of the included studies.
(DOCX)

Table S2 Summary of the included studies on herbal medicines for Parkinson's disease.
(DOCX)
