# Supplementary material for: Herbal Medicines for Parkinson's Disease: A Systematic Review of Randomized Controlled Trials
Source: PLoS One. 2012 May 15;7(5):e35695. doi: 10.1371/journal.pone.0035695 (PMC3352906; doi:10.1371/journal.pone.0035695)
Supplement: Table S1 — The preparation of the herbal medicines of the included studies. (DOCX) [file pone.0035695.s001.docx]

**Table S1. The preparation of the herbal medicines of the included studies**

| **Name of herbal medicine** | **Study ID** | **Preparation** | **Composition** |
| --- | --- | --- | --- |
| **Banisteriopsis Caapi extract** | Serrano 2001 [19] | Solution | Liquid extract of Banisteriopsis caapi vine |
| **Bushenpingchan fang** | [Cui 2003](Herbal%20medicines.xls#RANGE!_ENREF_56) [20] | Decoction | Herbal formula composed of 6 herbs: Polygoni Multiflori Radix 20g, Capreoli Cornu 1g, Gastrodiae Rhizoma 10g, Uncariae Ramulus et Uncus 15g, Bupleuri Radix 12g and Magnoliae Cortex 15g |
| **Bushenyanggan fangyao** | [Zhao 2007](Herbal%20medicines.xls#RANGE!_ENREF_3) [21] | Decoction | Herbal formula composed of 7 herbs: Cistanches Herba 10 g, Rehmanniae Radix Preparat 10 g, Corni Fructus 6 g, Angelica Sinensis Radix 10 g, Asparagi Radix 10 g, Paeoniae Radix Alba 10 g and Morindae Radix 10 g |
| **Budushujingxifeng tang** | [Liu 1994](Herbal%20medicines.xls#RANGE!_ENREF_63) [22] | Decoction | Herbal formula composed of 14 herbs: Astragali Radix, Rehmanniae Radix Preparat, Angelica Sinensis Radix, Paeoniae Radix Alba, Salviae Miltiorrhizae Radix, Spatholobi Caulis, Gastrodiae Rhizoma, Pheretimae Corpus, Bombycis Corpus cum Batryticatus, Chaenomelis Fructus, Puerariae Radix, Gazellae Cornu, Gynostemmae Herba and Hordei Fructus Germiniatus |
| **Bushenhuoxie recipe** | [Dou 2006](Herbal%20medicines.xls#RANGE!_ENREF_42) [23] | Decoction | Herbal formula composed of 8 herbs: Polygoni Multiflori Radix, Corni Fructus, Cistanches Herba, Lycii Fructus, Salviae Miltiorrhizae Radix, Acori Graminei Rhizoma, Scolopendra Corpus and Polygalae Radix |
| **Cannador capsule** | [Carroll 2004](Herbal%20medicines.xls#RANGE!_ENREF_69) [24] | Capsule | Extract of Cannabis sativa |
| **Chaihushugan san** | Yang 2010 [25] | Decoction | Herbal formula composed of 7 herbs: Bupleuri Radix, Cyperi Rhizoma, Ligustici Rhizoma, Paeoniae Radix Alba, Hirudo, Pheretimae Corpus and Glycyrrhizae Radix Praeparata |
| **Chuzhan tang** | [Zhang 2009](Herbal%20medicines.xls#RANGE!_ENREF_24) [26] | Decoction | Herbal formula composed of 12 herbs: Scorpio 6g, Bombycis Corpus cum Batryticatus 10g, Pheretimae Corpus 15g, Scolopendra Corpus 2 pieces, Uncariae Ramulus et Uncus 15g, Gastrodiae Rhizoma 10g, Cicadae Periostracum 10g, Lycii Fructus 15g, Angelica Sinensis Radix 15g, Paeoniae Radix Alba 30g, Ligustici Rhizoma 10g and Carthami Flos 10g |
| **Dingzhen tang_1** | [Zhang 2008](Herbal%20medicines.xls#RANGE!_ENREF_33) a [27]  [Zhang 2006](Herbal%20medicines.xls#RANGE!_ENREF_47) [28] | Decoction | Herbal formula composed of 14 herbs: Rehmanniae Radix Preparat 12g, Rehmanniae Radix 12g, Angelica Sinensis Radix 12g, Paeoniae Radix Alba 12g, Ligustici Rhizoma 9g, Astragali Radix 9g, Atractylodis Rhizoma Alba 9g, Gastrodiae Rhizoma 6g, Gentianae Macrophyllae Radix 6g, Saposhnikovia Radix 5g, Schizonepetae Spica 5g, Clematidis Radix 3g, Scorpio 3g and Asari Herba Cum Radix 3g |
| **Dingzhen tang_2** | [Zhu 2009](Herbal%20medicines.xls#RANGE!_ENREF_23) [29] | Decoction | Herbal formula composed of 17 herbs: Gastrodiae Rhizoma, Gentianae Macrophyllae Radix, Scorpio, Rehmanniae Radix Preparat, Rehmanniae Radix, Angelica Sinensis Radix, Ligustici Rhizoma, Paeoniae Radix, Saposhnikovia Radix, Schizonepetae Spica, Atractylodis Rhizoma Alba, Astragali Radix, Clematidis Radix, Cannabis Fructus, Cistanches Herba, Fossilia Ossis Mastodi and Ostreae Concha |
| **Dingzhen tang_3** | [Teng 2000](Herbal%20medicines.xls#RANGE!_ENREF_61) [30] | Decoction | Herbal formula composed of 9 herbs: Scorpio 6g, Bombycis Corpus cum Batryticatus 10g, Pheretimae Corpus 12g, Gastrodiae Rhizoma 10g, Uncariae Ramulus et Uncus 12g, Angelica Sinensis Radix 12g, Paeoniae Radix Alba 20g, Ligustici Rhizoma 10g and Salviae Miltiorrhizae Radix 15g |
| **Dingzhen tang_4** | [Ai 2004](Herbal%20medicines.xls#RANGE!_ENREF_52) [31] | Decoction | Herbal formula composed of 11 herbs: Gazellae Cornu 30g, Fossilia Ossis Mastodi 15g, Ostreae Concha 15g, Angelica Sinensis Radix 15g, Ligustici Rhizoma 15g, Leonuri Herba 20g, Gastrodiae Rhizoma 20g, Bombycis Corpus cum Batryticatus 15g, Hordei Fructus Germiniatus 30g, Mori Folium 15g and Tribuli Fructus 15g |
| **Dingzhan yin** | [Gu 2002](Herbal%20medicines.xls#RANGE!_ENREF_58) [32] | Decoction | Herbal formula composed of 9 herbs: Polygoni Multiflori Radix 20g, Taxilli Ramulus 15g, Ligustici Rhizoma 15g, Ostreae Concha 30g, Arisaema Praeparatus cum Bile 10g, Euonymi Caulis Suberatum 10g, Paeoniae Radix Alba 30g, Uncariae Ramulus et Uncus 30g and Scorpio 3g |
| **Fufangjangzhan wan** | [Yuan 2005](Herbal%20medicines.xls#RANGE!_ENREF_46) [33] | Pill | A hospital made preparation composed of 13 herbs: Rehmanniae Radix Preparat, Rehmanniae Radix, Paeoniae Radix Rubra, Paeonia albiflora, Angelica Sinensis Radix, Fossilia Ossis Mastodi, Ostreae Concha, Polygoni Multiflori Radix, Lycii Fructus, Spatholobi Caulis, Dendrobii Herba), Achyranthis Radix and Salviae Miltiorrhizae Radix |
| **Guilingpaan capsule** | Zhao 2009 [34] | Capsule | Mainly composed of Chinemydis Plastrum and Gazellae Cornu |
| **Guilingpaan wan** | [Chang 2008](Herbal%20medicines.xls#RANGE!_ENREF_30) [35]  [Jiang 2009](Herbal%20medicines.xls#RANGE!_ENREF_17) [36]  [Wang 2005](Herbal%20medicines.xls#RANGE!_ENREF_39) a [37] | Pill | A hospital made preparation composed of 5 herbs: Chinemydis Gelatina, Gazellae Cornu, Scorpio, Clematidis Radix and Magnoliae Cortex |
| **Jianpiyishen formula** | [Yang 2008](Herbal%20medicines.xls#RANGE!_ENREF_34) [38] | Decoction | Herbal formula composed of 15 herbs: Corni Fructus 15g, Dioscoreae Rhizoma 15g, Ligustri Lucidi Fructus 15g, Eucommiae Cortex 15g, Poria(Hoelen) 15g, Lycii Fructus 12g, Morindae Radix 12g, Schizandrae Fructus 12g, Rubi Fructus 12g, Astragali Radix 20g, Atractylodis Rhizoma Alba 20g, Paeoniae Radix Alba 20g, Polygoni Multiflori Radix 20g, Taxilli Ramulus 10g, Gastrodiae Rhizoma 10g |
| **Jiaweidadingfeng zhu** | [Ming 2010](Herbal%20medicines.xls#RANGE!_ENREF_12) [39] | Decoction | Herbal formula composed of 13 herbs: Rehmanniae Radix, Asini Gelatinum, Paeoniae Radix Alba, Liriopes Radix, Corni Fructus, Chinemydis Plastrum, Trionycis Carapax, Ostreae Concha, Cannabis Fructus, Schizandrae Fructus, Scorpio, Pheretimae Corpus and Glycyrrhizae Radix Praeparata |
| **Jiaweiguizhijiagegen tang** | [Lian 2008](Herbal%20medicines.xls#RANGE!_ENREF_48) [40] | Decoction | Herbal formula composed of 6 herbs: Cinnamomi Ramulus 15g, Paeoniae Radix Alba 15g, Puerariae Radix 40g, Zizyphi Fructus 20g, Glycyrrhizae Radix Praeparata 5g and Rhei Rhizoma 15-20g |
| **Kangpa granule** | [Zhao 2003](Herbal%20medicines.xls#RANGE!_ENREF_55) [41] | Granule | Chinese patient medicine: no information on the composition available |
| **Kangzhanning** | [Yang 2009](Herbal%20medicines.xls#RANGE!_ENREF_25) [42] | Decoction | A hospital made preparation composed of 13 herbs: Gastrodiae Rhizoma 15g, Paeoniae Radix Alba 20g, Chinemydis Plastrum 15g, Glycyrrhizae Radix Praeparata 9g, Astragali Radix 15g, Magnoliae Cortex 15g, Poria(Hoelen) 12g, Ligustici Rhizoma 6g, Spatholobi Caulis 15g and Angelica Sinensis Radix 12g |
| **Kangzhenzhijing capsule** | [Bao 2001](Herbal%20medicines.xls#RANGE!_ENREF_60) [43] | Capsule | A hospital made preparation composed of 14 herbs: Polygoni Multiflori Radix, Cistanches Herba, Salviae Miltiorrhizae Radix, Paeoniae Radix Alba, Gastrodiae Rhizoma, Scorpio, Lycii Fructus, Tokoro Rhizoma, Pheretimae Corpus, Agkistrodon, Astragali Radix, Spatholobi Caulis, Angelica Sinensis Radix and Chaenomelis Fructus |
| **Kanli tang** | [GUO 2004](Herbal%20medicines.xls#RANGE!_ENREF_51) [44] | Decoction | Herbal formula composed of 13 herbs: Chinemydis Plastrum 15g, Bombycidae 10g, Rehmanniae Radix 15g, Cicadae Periostracum 12g, Rhizoma Homalomenae 12g, Chaenomelis Fructus 12g, Acanthopanacis Cortex 15g, Carthami Flos 0.5g, Dragon's Teeth 15g, Bambusae Caulis In Taeniam 12g, Scolopendra Corpus, Agkistrodon and Glycyrrhizae Radix Praeparata 6g |
| **Lemai granule** | [Luo 2001](Herbal%20medicines.xls#RANGE!_ENREF_59) [45] | Granule | A hospital made preparation: : no information on the composition available |
| **Naokangning capsule** | [Kim 2004](Herbal%20medicines.xls#RANGE!_ENREF_13) [46] | Capsule | A hospital made preparation composed of 5 herbs: Polygoni Multiflori Radix, Gastrodiae Rhizoma, Uncariae Ramulus et Uncus, Ostreae Concha and Ligustici Rhizoma |
| **Nuzhenyangyin granule** | [Hu 2003](Herbal%20medicines.xls#RANGE!_ENREF_2) [47] | Granule | A hospital made preparation composed of 12 herbs: Ligustri Lucidi Fructus, Paeoniae Radix Alba, Uncariae Ramulus et Uncus, Ligustici Rhizoma, Magnoliae Cortex, Liriopes Radix, Bombycis Corpus cum Batryticatus, Angelica Sinensis Radix, Trichosanthis Radix, Atractylodis Rhizoma Alba and Glycyrrhizae Radix |
| **Pabing formula 1** | [Sun 2005](Herbal%20medicines.xls#RANGE!_ENREF_7) [48]  [Zhang 2008](Herbal%20medicines.xls#RANGE!_ENREF_9) b [49] | Decoction | Herbal formula composed of 10 herbs: Mume Fructus 10g, Angelica Sinensis Radix 10g, Corni Fructus 6g, Paeoniae Radix Alba 10g, Rehmanniae Radix Preparat 10g, Puerariae Radix 10g, Coptidis Rhizoma 3g, Ligustici Rhizoma 6g, Acori Graminei Rhizoma 6g and Glycyrrhizae Radix Praeparata 3g |
| **Pabing formula 2** | [Fan 2006](Herbal%20medicines.xls#RANGE!_ENREF_11) [50]  [Zhang 2008](Herbal%20medicines.xls#RANGE!_ENREF_9) b [49] | Decoction | Herbal formula composed of 10 herbs: Mume Fructus 10g, Corni Fructus 6g, Paeoniae Radix Alba 10g, Rehmanniae Radix Preparat 10g, Puerariae Radix 10g, Coptidis Rhizoma 3g, Ligustici Rhizoma 6g, Gastrodiae Rhizoma 6g, Acori Graminei Rhizoma 6g and Glycyrrhizae Radix Praeparata 3g |
| **Pabing formula 3** | [Zheng 2006](Herbal%20medicines.xls#RANGE!_ENREF_43) [51] | Decoction | Herbal formula composed of 8 herbs: Mume Fructus 10g, Coptidis Rhizoma 3g, Cinnamomi Ramulus 6g, Codonopsis Pilosulae Radix 10g, Angelica Sinensis Radix 10g, Ligustici Rhizoma 6g, Acori Graminei Rhizoma 6g and Glycyrrhizae Radix Praeparata 3g |
| **Peibuganshen recipe** | [Chen 1999](Herbal%20medicines.xls#RANGE!_ENREF_62) [52] | Decoction | Individualized herbal formula, mainly composed of 5 herbs: Polygoni Multiflori Radix, Cistanches Herba, Lycii Fructus, Gastrodiae Rhizoma and Uncariae Ramulus et Uncus |
| **Qingxinhuatan tang** | [An 2009](Herbal%20medicines.xls#RANGE!_ENREF_20) [53] | Decoction | Herbal formula composed of 9 herbs: Nelumbinis Plumula 9g, Coptidis Rhizoma 5g, Polygalae Radix 6g, Arisaema Praeparatus cum Bile 9g, Acori Graminei Rhizoma 12g, Notoginseng Radix 6g, Atractylodis Rhizoma Alba 9g, Bambusae Caulis In Taeniam 9g and Styrax Liquides 6g |
| **Rougantongluo tang** | [Wu 2008](Herbal%20medicines.xls#RANGE!_ENREF_37) a [54] | Decoction | Herbal formula, mainly composed of 12 herbs: Polygoni Multiflori Radix 15g, Ligustri Lucidi Fructus 30g, Mori Fructus 30g, Lycii Fructus 30g, Rehmanniae Radix Preparat 10g, Paeoniae Radix Alba 30g, Salviae Miltiorrhizae Radix 30g, Puerariae Radix 30g, Notoginseng Radix 3g, Tribuli Fructus 15g, Bombycis Corpus cum Batryticatus 10g and Scorpio 3g |
| **Shudipingzhan tang (1) plus Xiewu capsule (2)** | [Yuan 2010](Herbal%20medicines.xls#RANGE!_ENREF_8) [55] | (1) Granule | (1) A hospital made preparation composed of 8 herbs: Rehmanniae Radix Preparat 15g, Lycii Fructus 15g, Taxilli Ramulus 20g, Gastrodiae Rhizoma 15g, Bombycis Corpus cum Batryticatus 10g, Zedoariae Rhizoma 15g, Paeoniae Radix Alba 30g and Arisaematis Rhizoma 15g |
|  |  | (2) Capsule | (2) A hospital made preparation composed of 2 herbs: Scorpio and Scolopendra Corpus |
| **Shujinjiedu formula_1** | [Wang 2009](Herbal%20medicines.xls#RANGE!_ENREF_19) a [56] | Decoction | Herbal formula, mainly composed of 5 herbs: Spatholobi Caulis, Rehmanniae Radix Preparat, Scorpio, Paeoniae Radix Alba and Smilacis Glabrae Rhizoma |
| **Shujinjiedu formula_2** | [Wang 2009](Herbal%20medicines.xls#RANGE!_ENREF_16) b [57] | Decoction | Herbal formula, mainly composed of 5 herbs: Chinemydis Plastrum, Hirudo, Paeoniae Radix Alba, Bombycis Corpus cum Batryticatus and Salviae Miltiorrhizae Radix |
| **Tongxinluo capsule (1) plus Liuweidihuang wan (2)** | [Shen 2008](Herbal%20medicines.xls#RANGE!_ENREF_27) [58] | (1) Capsule | (1) Chinese patient medicine: no information on the composition available |
|  |  | (2) Pill | (2) Chinese patient medicine composed of 6 herbs: Rehmanniae Radix Preparat, Dioscoreae Rhizoma, Corni Fructus, Alismatis Rhizoma, Poria(Hoelen) and Moutan Cortex |
| **Wuhuzhuifeng san** | [Liang 2008](Herbal%20medicines.xls#RANGE!_ENREF_31) [59] | Granule | A commercial herbal preparation, mainly composed of 5 herbs: Cicadae Periostracum, Arisaematis Rhizoma, Gastrodiae Rhizoma, Scorpio and Bombycis Corpus cum Batryticatus |
| **Xifengdingzhan tang** | [Li 2008](Herbal%20medicines.xls#RANGE!_ENREF_65) [60] | Decoction | Herbal formula composed of 7 herbs: Chinemydis Plastrum 15g, Polygoni Multiflori Radix 12g, Gastrodiae Rhizoma 10g, Bombycis Corpus cum Batryticatus 9g, Acori Graminei Rhizoma 9g, Ligustici Rhizoma 10g and Paeoniae Radix Alba 10g |
| **Xifengdingzhan wan** | [Ma 2005](Herbal%20medicines.xls#RANGE!_ENREF_38) [61]  [Ma 2008](Herbal%20medicines.xls#RANGE!_ENREF_28) a [62]  [Ma 2008](Herbal%20medicines.xls#RANGE!_ENREF_29) b [63]  [Cheng 2007](Herbal%20medicines.xls#RANGE!_ENREF_41) [64] | Pill | A hospital made preparation, mainly composed of 7 herbs: Polygoni Multiflori Radix, Chinemydis Plastrum, Gastrodiae Rhizoma, Bombycis Corpus cum Batryticatus, Acori Graminei Rhizoma, Ligustici Rhizoma and Paeoniae Radix Alba |
| **Xifengzhizhan tang** | [Lu 2009](Herbal%20medicines.xls#RANGE!_ENREF_21) [65] | Decoction | Herbal formula, mainly composed of 7 herbs: Paeoniae Radix Alba 30g, Angelica Sinensis Radix 12g, Gastrodiae Rhizoma 9g, Rehmanniae Radix 15g, Corni Fructus 9g, Chinemydis Plastrum 9g, Lycii Fructus 15g, Anemarrhenae Rhizoma 12g, Achyranthis Radix 12g and Bombycis Corpus cum Batryticatus 9g |
| **Yangganxifeng recipe** | [Wang 2008](Herbal%20medicines.xls#RANGE!_ENREF_32) a [66] | Decoction | Herbal formula composed of 16 herbs: Rehmanniae Radix Preparat 24g, Dioscoreae Rhizoma 12g Lycii Fructus 15g, Corni Fructus 15g, Achyranthis Radix 15g, Cuscutae Semen 15g, Chinemydis Plastrum 15g, Cervi Cornus Colla 15g, Salviae Miltiorrhizae Radix 15g, Carthami Flos 10g, Polygoni Multiflori Radix 15g, Astragali Radix 15g, Gypsum Fibrosum 21g, Scrophulariae Radix 15g, Veratri Nigri Rhizoma et Radix 21g and Glycyrrhizae Radix 3g |
| **Yiguan jian plus Dabuyin wan** | [Li 2009](Herbal%20medicines.xls#RANGE!_ENREF_18) [67] | Decoction | Herbal formula composed of 8 herbs: Chinemydis Plastrum 30g, Rehmanniae Radix Preparat 30g, Adenophorae Radix 10g, Liriopes Radix 10g, Lycii Fructus 15g, Angelica Sinensis Radix 10g, Anemarrhenae Rhizoma 12g and Phellodendri Cortex 10g |
| **Yiyuan yin** | [Chen 2009](Herbal%20medicines.xls#RANGE!_ENREF_15) [68] | Decoction | Herbal formula, mainly composed of 15 herbs: Rehmanniae Radix Preparat 15g, Corni Fructus 10g, Polygonati Rhizoma 10g, Angelica Sinensis Radix 10g, Paeoniae Radix Alba 15g, Astragali Radix 15g, Poria(Hoelen) 15g, Gastrodiae Rhizoma 10g, Bombycis Corpus cum Batryticatus 10g, Ostreae Concha 20g, Cistanches Herba 12g, Cinnamomi Cortex Spissus 1.5g, Taxilli Ramulus 15g, Citrus medica L.var.sarcodactylis 10g and Glycyrrhizae Radix Praeparata 6g |
| **Yizhan tang** | [Yang 2002](Herbal%20medicines.xls#RANGE!_ENREF_57) [69] | Decoction | Herbal formula composed of 7 herbs: Corni Fructus, Acori Graminei Rhizoma, Epimedi Herba, Cistanches Herba, Lycii Fructus, Salviae Miltiorrhizae Radix and Scolopendra Corpus |
| **Zengxiao Anshen Zhichan 2 recipe** | [Pan 2009](Herbal%20medicines.xls#RANGE!_ENREF_1) [70] | Capsule | A herbal preparation composed of 14 herbs: Rehmanniae Radix Preparat, Corni Fructus, Fossilia Ossis Mastodi, Asparagi Radix, Paeoniae Radix Alba, Chinemydis Plastrum, Cistanches Herba, Puerariae Radix, Arisaema Praeparatus cum Bile, Scorpio, Salviae Miltiorrhizae Radix, Pheretimae Corpus, Acori Graminei Rhizoma and Curcumae Longae Rhizoma |
| **ZhenChan shu** | [Feng 2002](Herbal%20medicines.xls#RANGE!_ENREF_70) [71] | Capsule | A herbal preparation composed of 4 herbs: Paeoniae Radix Alba, Glycyrrhizae Radix Praeparata, Puerariae Radix and Magnoliae Cortex |
| **Zhenchan tang** | [Wu 2008](Herbal%20medicines.xls#RANGE!_ENREF_35) b [72] | Decoction | Herbal formula, mainly composed of 4 herbs: Chinemydis Plastrum, Hirudo, Bombycis Corpus cum Batryticatus and Salviae Miltiorrhizae Radix |
| **Zhichanshudu tang** | [Wang 2008](Herbal%20medicines.xls#RANGE!_ENREF_36) b [73] | Decoction | Herbal formula composed of 10 herbs: Chinemydis Plastrum 20g, Scrophulariae Radix 20g, Paeoniae Radix Alba 15g, Eucommiae Cortex 15g, Gastrodiae Rhizoma 15g, Achyranthis Radix 15g, Lycii Fructus 15g, Rehmanniae Radix Preparat 15g, Liriopes Radix 15g and Glycyrrhizae Radix 15g |
| **Zhizhan tang** | [Wang 2010](Herbal%20medicines.xls#RANGE!_ENREF_10) [74] | Decoction | Herbal formula composed of 7 herbs: Anemarrhenae Rhizoma 9g, Paeoniae Radix Alba 30g, Uncariae Ramulus et Uncus 18g, Astragali Radix 20g, Salviae Miltiorrhizae Radix 20g, Cimicifugae Rhizoma 9g and Rhei Rhizoma 9g |
| **Zibuganshen recipe** | [Shen 2006](Herbal%20medicines.xls#RANGE!_ENREF_44) [75] | Decoction | Herbal formula, mainly composed of 16 herbs: Rehmanniae Radix Preparat 10g, Lycii Fructus 12g, Taxilli Ramulus 20g, Gastrodiae Rhizoma 15g, Uncariae Ramulus et Uncus 15g, Bombycis Corpus cum Batryticatus 9g, Pheretimae Corpus 12g, Ligustici Rhizoma 12g, Zedoariae Rhizoma 9g, Paeoniae Radix Alba 20g, Arisaematis Rhizoma 15g, Scolopendra Corpus 1.5g and Scorpio 1.5g |
| **Zibuganshen、Huoxiexifeng recipe** | [Jiang 2003](Herbal%20medicines.xls#RANGE!_ENREF_54) [76] | Decoction | Herbal formula, mainly composed of 12 herbs: Polygoni Multiflori Radix 30g, Rehmanniae Radix 10g, Rehmanniae Radix Preparat 10g, Scrophulariae Radix 20g, Salviae Miltiorrhizae Radix 20g, Paeoniae Radix Rubra 15g, Paeoniae Radix Alba 15g, Gastrodiae Rhizoma 10g, Uncariae Ramulus et Uncus 15g, Fossilia Ossis Mastodi 30g, Ostreae Concha 30g and Tribuli Fructus 10g |
| **Ziyinxifeng granule** | [Tang 2005](Herbal%20medicines.xls#RANGE!_ENREF_40) [77] | Granule | A commercial herbal preparation, composed of 12 herbs: Chinemydis Plastrum 20g, Salviae Miltiorrhizae Radix 20g, Cervi Cornus Degelatinatum 12g, Corni Fructus 12g, Dioscoreae Rhizoma 12g, Eucommiae Cortex 12g, Ligustici Rhizoma 12g, Rehmanniae Radix Preparat 24g, Margaritifera Usta Concha 30g, Paeoniae Radix Alba 15g, Gastrodiae Rhizoma 15g and Glycyrrhizae Radix Praeparata 15g |
| **Ziyinxifenghuoxie tang** | [Wang 2004](Herbal%20medicines.xls#RANGE!_ENREF_53) [78] | Decoction | Herbal formula composed of 5 herbs: Rehmanniae Radix Preparat 24g, Corni Fructus 12g, Paeoniae Radix Alba 15g, Gastrodiae Rhizoma 15g and Ligustici Rhizoma 12g |
| **Herbal recipe_no name** | [Cui 2004](Herbal%20medicines.xls#RANGE!_ENREF_50) [79] | Decoction | Herbal formula composed of 20 herbs: Margaritifera Usta Concha 60g, Gazellae Cornu 1.5g, Uncariae Ramulus et Uncus 15g, Paeoniae Radix Alba 15g, Angelica Sinensis Radix 6g, Paeoniae Radix Rubra 10g, Ligustici Rhizoma 10g, Persicae Semen 6g, Carthami Flos 6g, Spatholobi Caulis 15g, Bombycis Corpus cum Batryticatus 10g, Pheretimae Corpus 15g, Scorpio 3g, Scolopendra Corpus 1 piece, Rehmanniae Radix Preparat 15g, Lycii Fructus 15g, Corni Fructus 15g, Polygoni Multiflori Radix 15g, Eucommiae Cortex 15g and Taxilli Ramulus 15g |
| **Individualized herbal recipe** | [Qiu 1998](Herbal%20medicines.xls#RANGE!_ENREF_49) [80] | Decoction | Five different herbal recipes used according to the five Chinese syndrome differentiation |
| **Individualized herbal recipe** | [Ma 2003](Herbal%20medicines.xls#RANGE!_ENREF_6) [81] | Decoction | Four different herbal recipes used according to the four Chinese syndrome differentiation |
|  |  |  | (1) Dadingfeng zhu: Herbal formula, mainly composed of 10 herbs (Paeoniae Radix Alba 30g, Rehmanniae Radix 30g, Schizandrae Fructus 15g, Dioscoreae Rhizoma 15g, Liriopes Radix 15g, Chinemydis Plastrum 30g, Gastrodiae Rhizoma 15g, Corni Fructus 12g, Moutan Cortex 10g and Scorpio10g) |
|  |  |  | (2) Rencanyangrong tang he tianmagouteng tang: Herbal formula, mainly composed of 10 herbs (Pseudostellariae Radix 20g, Poria(Hoelen) 10g, Atractylodis Rhizoma Alba 10g, Angelica Sinensis Radix 15g, Paeoniae Radix Alba 30g, Rehmanniae Radix Preparat 15g, Gastrodiae Rhizoma 10g, Uncariae Ramulus et Uncus 15g, Astragali Radix 15g and Spatholobi Caulis 20g) |
|  |  |  | (3) Huanglianwendan tang he tianmagouteng tang: Herbal formula, mainly composed of 12 herbs (Coptidis Rhizoma 10g, Scutellariae Radix 10g, Arisaema Praeparatus cum Bile 12g, Pinelliae Rhizoma 10g, Fructus Trichosanthis 15g, Acori Calami Rhizoma 15g, Gastrodiae Rhizoma 10g, Uncariae Ramulus et Uncus 15g, Haliotidis Concha 30g, Paeoniae Radix Alba 30g, Chaenomelis Fructus 15g and Scolopendra Corpus 2 pieces) |
|  |  |  | (4) Xiefuzhuyu tang he tianmagouteng yin: Herbal formula, mainly composed of 11 herbs (Rehmanniae Radix 15g, Paeoniae Radix Alba 30g, Paeoniae Radix Rubra 15g, Salviae Miltiorrhizae Radix 30g, Gastrodiae Rhizoma 10g, Pheretimae Corpus 10g, Uncariae Ramulus et Uncus 12g, Aurantii Fructus Pericarpium 10g, Bupleuri Radix 15g, Achyranthis Radix 15g and Lycopodii Herba 20g) |
| **Individualized herbal recipe** | [Wang 2005](Herbal%20medicines.xls#RANGE!_ENREF_45) b [82] | Decoction | Six different herbal recipes used according to the six Chinese syndrome differentiation |
