# Supplementary material for: Herbal Medicines for Parkinson's Disease: A Systematic Review of Randomized Controlled Trials
Source: PLoS One. 2012 May 15;7(5):e35695. doi: 10.1371/journal.pone.0035695 (PMC3352906; doi:10.1371/journal.pone.0035695)
Supplement: Table S2 — Summary of the included studies on herbal medicines for Parkinson's disease. (DOCX) [file pone.0035695.s002.docx]

**Table S2. Summary of the included studies on herbal medicines for Parkinson’s disease**

| **First author Year [Ref]** | **Disease duration (mean, SD)/Severity (mean, SD)** | **Intervention for the treatment group (treatment period, number)** | **Intervention for the control group (number)** | **Outcome** | **Time of the last follow-up** | **Effect size estimates (RR or MD)** | **Risk of bias** |  |  |
| --- | --- | --- | --- | --- | --- | --- | --- | --- | --- |
| Serrano 2001[19] | 24.9, 10.3 m/ n.r. | (A)Banisteriopsis Caapi extract (once, n=15) | (B)Placebo (n=15) | (1)Total UPDRS score | 4hrs | (1)MD -27.40 [-28.19, -26.61] | U,U,L,L,U,L |  |  |
| Cui 2003[20] | 4.15, 4.72 y/ total UPDRS score 58.15, 47.20 | (A)Bushenpingchan formula + (B) (bid for 3 months, n=35) | (B)Madopar 130 to2000mg (n=35) | (1)Global improvement of symptoms | 3 months | (1)RR 1.33 [0.90, 1.97] | U,U,H,U,U,L |  |  |
|  |  |  |  | (2)Total UPDRS score |  | (2)MD -3.30 [-16.79, 10.19] |  |  |  |
|  |  |  |  | (3)Dose of madopar |  | (3)MD -199.30 [-367.65, -30.95] |  |  |  |
| Zhao 2007[21] | 4.53, 0.44 y/  H-Y staging 1.8, 0.47 | (A)Bushenyanggan recipe + (B) (for 12 months, n=55) | (B)Levodopa (n=58) | (1)Total UPDRS score | 12 months | (1)MD -5.10 [-6.01, -4.19] | L,L,H,U,U,L |  |  |
|  |  |  |  | (2)H-Y staging |  | (2)MD -0.20 [-0.40, 0.00] |  |  |  |
|  |  |  |  | (3)Dose of levodopa |  | (3)MD -80.03 [-113.25, -46.81] |  |  |  |
| Liu 1994[22] | A:6y, B:5.8y/ n.r. | (A)Budushujingxifen tang + (B) (for 3 months, n=45) | (B)Madopar 375 to 1000mg, bromocriptine 2.5 to 20mg (n=30) | (1)Global improvement of symptoms | 3 months | (1)RR 1.21 [0.92, 1.57] | U,U,H,U,U,U |  |  |
| Dou 2006[23] | 11.4, 4.6 y/ n.r. | (A)Bushenhuoxie recipe + (B) (for 12 weeks, n=52) | (B)Madopar 500 to 600mg + bromocriptine 2.5 to 10mg (n=37) | (1)Global improvement of symptoms | 12 weeks | (1)RR 1.20 [0.95, 1.53] | L,U,H,L,U,L |  |  |
|  |  |  |  | (2)Dose of madopar |  | (2)MD -121.20 [-149.04, -93.36] |  |  |  |
|  |  |  |  | (3)Dose of bromocriptine |  | (3)MD -2.48 [-2.70, -2.26] |  |  |  |
|  |  |  |  | (4)Nausea and vomiting (NMP) |  | (4)RR 0.40 [0.20, 0.81] |  |  |  |
|  |  |  |  | (5)Constipation (NMP) |  | (5)RR 0.11 [0.03, 0.46] |  |  |  |
| Carroll 2004[24] | 14 y/ H-Y staging 3 | (A)Cannador capsule + western drug (for 4 weeks, n=9) | (B)Placebo + western drug (n=8) | (1)Dyskinesia score | 4 weeks | (1)MD 0.56 [-1.11, 2.23] | L,L,L,L,U,U |  |  |
| Yang 2010 [25] | 4.35, 2.19 y/ n.r. | (A)Chaihushugan san + (B) (for 8 weeks,n=30) | (B)Paroxetine 20mg (n=30) | (1)Global improvement of depression | 8weeks | (1)RR 1.29 [0.99, 1.67] | U,U,U,L,U,L |  |  |
|  |  |  |  | (2)HAM-D |  | (2)MD -4.10 [-5.17, -3.03] |  |  |  |
|  |  |  |  | (3)Adverse event rate |  | (3)RR 0.48 [0.31, 0.74] |  |  |  |
| Zhang 2009[26] | n.r./ Webster scale 14.77, 2.94 | (A)Chuzhan tang + (B) (for 3 months, n=34) | (B)Levodopa 250 mg, trastal 150 mg (n=18) | (1)Global improvement of symptoms | 3 months | (1)RR 1.30 [0.97, 1.76] | U,U,H,U,U,L |  |  |
|  |  |  |  | (2)Webster scale |  | (2)MD -1.42 [-3.06, 0.22] |  |  |  |
| Zhang 2008 a [27] | <6 m/ H-Y staging 1 to 2.5 | (A)Dingzhen tang_1 + (B) (1 session: 3 weeks, total 3 sessions, n=30) | (B)Madopar 250 to 325mg bid (n=30) | (1)Global improvement of symptoms | 3 months | (1)RR 1.29 [0.99, 1.67] | U,U,H,U,U,L |  |  |
|  |  |  |  | (2)UPDRS I subscore |  | (2)MD -1.23 [-2.24, -0.22] |  |  |  |
|  |  |  |  | (3)UPDRS II subscore |  | (3)MD -1.20 [-2.73, 0.33] |  |  |  |
|  |  |  |  | (4)UPDRS III subscore |  | (4)MD -1.90 [-3.40, -0.40] |  |  |  |
|  |  |  |  | (5)UPDRS IV subscore |  | (5)MD -0.04 [-0.63, 0.55] |  |  |  |
| Zhang 2006[28] | 3.85, 2.60 y/ H-Y staging 2.02, 0.65 | (A)Dingzhen tang_1 + madopar 250 to 500 mg (1 session: 3 weeks, total 3 sessions, n=30) | (B)Placebo + madopar 250 to 500 mg (n=30) | (1)Global improvement of symptoms | 3 months | (1)RR 1.29 [0.99, 1.67] | L,U,L,U,U,L |  |  |
|  |  |  |  | (2)UPDRS I subscore |  | (2)MD -0.74 [-1.72, 0.24] |  |  |  |
|  |  |  |  | (3)UPDRS II subscore |  | (3)MD -1.87 [-4.55, 0.81] |  |  |  |
|  |  |  |  | (4)UPDRS III subscore |  | (4)MD -3.57 [-7.00, -0.14] |  |  |  |
|  |  |  |  | (5)UPDRS IV subscore |  | (5)MD -1.16 [-2.49, 0.17] |  |  |  |
| Zhu 2009[29] | 3.40, 2.43 y/ H-Y staging 2.22, 0.68 | (A)Dingzhen tang_2 + (B)  (for 6 months, n=34) | (B)Conventional treatment  (n=31) | (1)UPDRS III subscore  (2)Constipation (NMP) | 6 months | (1)MD -4.30 [-7.79, -0.81]  (2)RR 0.48 [0.29, 0.78] | U,U,H,U,U,L |  |  |
| Teng 2000[30] | n.r./ n.r. | (A)Dingzhen tang_3 (for 3 months, n=32) | (B)Levodopa 750mg, artane 6mg, amantadine 300mg (n=30) | (1)Global improvement of symptoms | 3 months | (1)RR 1.00 [0.94, 1.06] | U,U,H,U,U,L |  |  |
| Ai 2004[31] | A:5.6y, B:5.9y/ n.r. | (A)Dingzhen tang_4 + (B) (for 3 months, n=23) | (B)Madopar 750mg, artane 2mg (n=21) | (1)Global improvement of symptoms | 3 months | (1)RR 1.22 [0.89, 1.67] | H,U,H,U,U,U |  |  |
|  |  |  |  | (2)Adverse event rate |  | (2)RR 0.39 [0.18, 0.83] |  |  |  |
| Gu 2002[32] | n.r./H-Y staging 2.47, 1.33 | (A)Dingzhan yin + (B) (for 6 months, n=30) | (B)Madopar 250mg (n=30) | (1)Global improvement of symptoms | 6 months | (1)RR 1.18 [0.91, 1.53] | U,U,H,U,U,L |  |  |
| Yuan 2005[33] | n.r./ n.r. | (A)Fufangjangzhan wan + (B) (for 30 days, n=60) | (B)Madopar (n=60) | (1)Global improvement of symptoms | 30 days | (1)RR 1.40 [1.16, 1.70] | U,U,H,U,U,L |  |  |
| Zhao 2009[34] | 4.58, 3.20 y/ H-Y staging 1.5 to 4 | (A)Guilingpaan capsule (for 12 weeks, n=28) | (B)Placebo (n=25) | (1)Global improvement of symptoms | 12 weeks | (1) 1) A:B RR 1.30 [0.83, 2.06] | L,U,L,U,U,L |  |  |
|  |  |  | (C)Levodopa + (A) (n=94) | (2)Total UPDRS score |  | 2) C:D RR 1.11 [0.92, 1.33] |  |  |  |
|  |  |  | (D)Levodopa + (B) (n=95) | (3)UPDRS II subscore |  | (2) 1) A:B MD -3.45 [-14.99, 8.09] |  |  |  |
|  |  |  |  | (4)UPDRS III subscore |  | 2) C:D MD -2.95 [-9.55, 3.65] |  |  |  |
|  |  |  |  | (5)Dose of levodopa |  | (3) C:D MD -1.17 [-3.42, 1.08] |  |  |  |
|  |  |  |  |  |  | (4) C:D MD -1.42 [-6.02, 3.18] |  |  |  |
|  |  |  |  |  |  | (5)C:D MD 12.38 [-41.50, 66.26] |  |  |  |
| Chang 2008[35] | 3.5, 1.40y/ total UPDRS score 63.42, 21.75 | (A)Guilingpaan wan + (B) (for 3 months, n=30) | (B)Madopar 250 to 375mg (n=30) | (1)Global improvement of symptoms | 3 months | (1)RR 1.45 [1.20, 1.77] | U,U,H,U,U,L |  |  |
|  |  |  |  | (2)Total UPDRS score |  | (2)MD -9.97 [-16.68, -3.25] |  |  |  |
|  |  |  |  | (3)Nausea & vomiting (NMP) |  | (3)RR 0.61 [0.33, 1.11] |  |  |  |
| Jiang 2009[36] | 4.83, 1.62 y/ H-Y staging 1.5 to 3 | (A)Guilingpaan wan + (B) (for 3 months, n=30) | (B)Madopar (n=30) | (1)Global improvement of symptoms | 3 months | (1)RR 1.45 [1.20, 1.77] | U,U,H,U,U,U |  |  |
|  |  |  |  | (2)Total UPDRS score |  | (2)MD -9.97 [-16.68, -3.25] |  |  |  |
|  |  |  |  | (3)Nausea (NMP) |  | (3)RR 0.61 [0.33, 1.11] |  |  |  |
|  |  |  |  | (4)Constipation(NMP) |  | (4)RR 0.44 [0.15, 1.29] |  |  |  |
| Wang 2005 a [37] | 2.93, 1.24 y/ UPDRS II+III subscore 31.21, 2.17 | (A)Guilingpaan wan + (B) (for 3 months, n=38) | (B)Madopar 187.5 mg (n=38) | (1)Global improvement of symptoms | 3 months | (1)RR 1.45 [1.20, 1.77] | U,U,H,U,U,L |  |  |
|  |  |  |  | (2)UPDRS II+ III Subscore |  | (2)MD -4.85 [-5.89, -3.81] |  |  |  |
|  |  |  |  | (3)Nausea & vomiting (NMP) |  | (3)RR 0.61 [0.33, 1.11] |  |  |  |
| Yang 2008[38] | 4.59, 3.01 y/ H-Y 2.16, 0.92 | (A)Jianpiyishen formula + (B) (for 8 weeks, n=32) | (B)Artane 4 to 6mg, madopar 125 to 600 mg (n=32) | (1)UPDRS II subscore | 8 weeks | (1)MD -5.08 [-8.84, -1.32] | U,U,H,U,U,L |  |  |
|  |  |  |  | (2)H-Y staging |  | (2)MD -0.25 [-0.68, 0.18] |  |  |  |
|  |  |  |  | (3)Adverse event rate |  | (3)RR 0.36 [0.13, 1.03] |  |  |  |
| Ming 2010[39] | n.r./ n.r. | (A)Jiaweidadingfeng zhu + Madopar (for 28 days, n=30) | (B)Placebo + madopar (n=30) | (1)Global improvement of symptoms | 28 days | (1)RR 3.83 [1.82, 8.05] | U,U,L,U,U,L |  |  |
|  |  |  |  | (2)UPDRS I subscore |  | (2)MD -0.09 [-1.11, 0.93] |  |  |  |
|  |  |  |  | (3)UPDRS II subscore |  | (3)MD -5.53 [-8.63, -2.43] |  |  |  |
|  |  |  |  | (4)UPDRSIIIsubscore |  | (4)MD -3.19 [-6.69, 0.31] |  |  |  |
| Lian 2008[40] | n.r./ H-Y staging 1.99, 0.73 | (A)Jiaweiguizhijiagegen tang + Madopar 375mg (1 session: 3 weeks, total 3 seesions, n=30) | (B)Placebo + madopar 375mg (n=30) | (1)UPDRS I subscore | 3 months | (1)MD -0.87 [-1.84, 0.10] | U,U,L,U,U,L |  |  |
|  |  |  |  | (2)UPDRS II subscore |  | (2)MD -2.03 [-4.70, 0.64] |  |  |  |
|  |  |  |  | (3)UPDRSIIIsubscore |  | (3)MD -2.93 [-6.20, 0.34] |  |  |  |
|  |  |  |  | (4)UPDRSIVsubscore |  | (4)MD -0.73 [-2.07, 0.61] |  |  |  |
| Zhao 2003[41] | n.r./ UPDRS IV subscore 12.06, 2.13 | (A)Kangpa granule + (B) (for 3 months, n=30) | (B)Madopar 750-2000mg (n=26) | (1)Global improvement of symptoms | 3 months | (1)RR 3.13 [1.79, 5.46] | U,U,H,U,U,U |  |  |
|  |  |  |  | (2)UPDRSIVsubscore |  | (2)MD -8.94 [-10.30, -7.58] |  |  |  |
| Yang 2009[42] | n.r./ n.r. | (A)Kangzhanning + (B) (for 2 months, n=24) | (B)Madopar(n=24) | (1)Total UPDRS score | 2 months | (1)MD -7.00 [-8.88, -5.12] | U,U,H,U,U,L |  |  |
|  |  |  |  | (2)Constipation(NMP) |  | (2)RR 0.50 [0.28, 0.88] |  |  |  |
| Bao 2001[43] | 4.80, 1.90 y/ Webster scale 14.05, 3.17 | (A)Kangzhenzhijing capsule (for 3 months, n=30) | (B)Levodopa, madopar, Artane  (n=29) | (1)Global improvement of symptoms | 3 months | (1)A:B RR 0.97 [0.83, 1.13] | U,U,H,U,U,L |  |  |
|  |  |  | (C)(A)+(B) (n=29) | (2)Webster scale |  | (2)A:B MD -1.80 [-3.40, -0.20] |  |  |  |
| Guo 2004[44] | 6.10, 2.80 y/ Webster scale 14.51, 3.45 | (A)Kanli tang (for 12 weeks, n=20) | (B)Madopar 500 to 1000 mg (n=20) | (1)Global improvement of symptoms | 12 weeks | (1)A:B RR 1.50 [0.79, 2.86] | U,U,H,U,U,L |  |  |
|  |  |  | (C)(A)+(B) (n=21) | (2)Webster scale |  | (2)A:B MD -2.40 [-4.29, -0.51] |  |  |  |
| Luo 2001[45] | n.r./ total UPDRS score 48.65, 7.98 | (A)Lemai granule + (B) (for 6 weeks, n=32) | (B)Madopar 500 to 1000mg, Vit B6 30 mg (n=20) | (1)Total UPDRS score | 6 weeks | (1)MD -5.40 [-10.05, -0.75] | U,U,H,U,U,L |  |  |
|  |  |  |  | (2)Adverse event rate |  | (2)RR 0.94 [0.17, 5.13] |  |  |  |
| Kim 2004[46] | 5.13, 3.14 y/ H-Y staging 2.4, 0.76 | (A)Naokangning capsule + madopar (for 3 months, n=30) | (B)Placebo+madopar (n=30) | (1)Global improvement of symptoms | 12 weeks | (1)RR 1.53 [1.02, 2.31] | L,U,L,U,U,L |  |  |
|  |  |  |  | (2)Total UPDRS score |  | (2)MD -6.00 [-11.24, -0.76] |  |  |  |
|  |  |  |  | (3)Dose of madopar |  | (3)MD -150.00 [-264.47, -35.53] |  |  |  |
| Hu 2003[47] | 5.92, 1.22 y/ n.r. | (A)Nuzhenyangyin granule + Madopar, Artane (for 2 months, n=30) | (B)Placebo+Madopar, Artane (n=30) | (1)Global improvement of symptoms | 2 months | (1)RR 1.53 [1.09, 2.16] | L,H,L,L,U,U |  |  |
| Sun 2005[48] | 5.37, 4.48 y/ H-Y staging 2.15, 0.83 | (A)Pabing formula 1 + (B) (for 3 months, n=22) | (B)Madopar250mg ( n=19) | (1)Global improvement of symptoms | 3 months | (1)RR 0.96 [0.84, 1.09] | L,U,H,U,U,L |  |  |
|  |  |  |  | (2)Total UPDRS score |  | (2)MD -9.04 [-17.11, -0.97] |  |  |  |
|  |  |  |  | (3)UPDRS I subscore |  | (3)MD -0.73 [-1.65, 0.19] |  |  |  |
|  |  |  |  | (4)UPDRS II subscore |  | (4)MD -4.42 [-8.10, -0.74] |  |  |  |
|  |  |  |  | (5)UPDRSIIIsubscore |  | (5)MD -4.16 [-8.59, 0.27] |  |  |  |
|  |  |  |  | (6)UPDRSIVsubscore |  | (6)MD -0.12 [-0.82, 0.58] |  |  |  |
| Zhang 2008 b [49] | 3.91, 2.16 y/ H-Y staging 2, 0.76 | (A)Pabing formula 1 or Pabing formula 2 + (B) (1 session: 3 weeks, total for 3 months, n=30) | (B)Madopar 250 to 500 mg (n=30) | (1)Global improvement of symptoms | 3 months | (1)RR 1.24 [0.94, 1.63 | U,U,H,U,U,U |  |  |
|  |  |  |  | (2)UPDRS I subscore |  | (2)MD -0.73 [-1.58, 0.12] |  |  |  |
|  |  |  |  | (3)UPDRS II subscore |  | (3)MD -1.46 [-3.95, 1.03] |  |  |  |
|  |  |  |  | (4)UPDRSIIIsubscore |  | (4)MD 0.59 [-2.21, 3.39] |  |  |  |
|  |  |  |  | (5)UPDRSIVsubscore |  | (5)MD -0.97 [-2.34, 0.40] |  |  |  |
| Fan 2006[50] | 4.95, 4.39 y/ H-Y staging 1.92, 0.63 | (A)Pabing formula 2 + (B) (for 3 months, n=30) | (B)Madopar 250mg (n=30) | (1)Global improvement of symptoms | 3 months | (1)RR 2.80 [1.15, 6.80] | L,L,H,U,U,L |  |  |
|  |  |  |  | (2)UPDRS I subscore |  | (2)MD -0.87 [-1.84, 0.10] |  |  |  |
|  |  |  |  | (3)UPDRS II subscore |  | (3)MD -2.03 [-4.70, 0.64] |  |  |  |
|  |  |  |  | (4)UPDRSIIIsubscore |  | (4)MD -2.93 [-6.20, 0.34] |  |  |  |
|  |  |  |  | (5)UPDRSIVsubscore |  | (5)MD -0.73 [-2.10, 0.64] |  |  |  |
| Zheng 2006[51] | >6 m/ H-Y staging 2, 0.76 | (A)Pabing formula 3 + madopar 250 to 500 mg (1 session: 3 weeks, total 3 sessions, n=30) | (B)Placebo+ madopar 250 to 500 mg (n=30) | (1)Global improvement of symptoms | 3 months | (1)RR 2.60 [1.06, 6.39] | U,U,L,U,U,L |  |  |
|  |  |  |  | (2)UPDRS I subscore |  | (2)MD -0.67 [-1.64, 0.30] |  |  |  |
|  |  |  |  | (3)UPDRS II subscore |  | (3)MD -2.24 [-5.21, 0.73] |  |  |  |
|  |  |  |  | (4)UPDRSIIIsubscore |  | (4)MD -3.23 [-6.72, 0.26] |  |  |  |
|  |  |  |  | (5)UPDRSIVsubscore |  | (5)MD -0.83 [-2.23, 0.57] |  |  |  |
| Chen 1999[52] | 6.2, 2.59 y/ Webster scale 14, 4.43 | (A)Peibuganshen recipe + (B) (for 3 months, n=30) | (B)Madopar, artane (n=30) | (1)Global improvement of symptoms | 3 months | (1)RR 1.89 [1.01, 3.55] | U,U,H,U,U,L |  |  |
|  |  |  |  | (2)Webster scale |  | (2)MD -3.10 [-5.00, -1.2] |  |  |  |
| An 2009[53] | 5.84, 1.20 y/ n.r. | (A)Qingxinhuatan tang (for 12 weeks, n=30) | (B)Madopar 250 to 750mg (n=30) | (1)Global improvement of symptoms | 12 weeks | (1)RR 1.33 [1.04, 1.72] | U,U,H,L,U,L |  |  |
| Wu 2008 a [54] | 5.98, 1.49 y/ n.r. | (A)Rougantongluo tang + (B) (for 1 month, n=36) | (B)Madopar (n=36) | (1)Global improvement of symptoms | 1 month | (1)RR 1.30 [0.98, 1.74] | U,U,H,L,U,L |  |  |
|  |  |  |  | (2) Adverse event rate |  | (2)RR 0.36 [0.14, 0.89] |  |  |  |
| Yuan 2010[55] | 7.39, 1.73y/ H-Y staging 2.75, 0.64 | (A)Shudipingzhan tang plus Xiewu capsule + (B) (twice per day for for 3 months, n=60) | (B)Levodopa ( n=60) | (1)Global improvement of symptoms | 3 months | (1)RR 2.27 [1.60, 3.23] | L,L,H,L,U,L |  |  |
|  |  |  |  | (2)UPDRS II subscore |  | (2)MD -1.72 [-3.32, -0.12] |  |  |  |
|  |  |  |  | (3)UPDRSIIIsubscore |  | (3)MD -2.48 [-4.34, -0.62] |  |  |  |
|  |  |  |  | (4)Dose of levodopa |  | (4)MD 6.90 [-39.42, 53.22] |  |  |  |
|  |  |  |  | (5)Nausea& epigastric pain (NMP) |  | (5)RR 1.40 [0.68, 2.90] |  |  |  |
|  |  |  |  | (6)Constipation (NMP) |  | (6)RR 0.88 [0.56, 1.38] |  |  |  |
| Wang 2009 a [56] | 5.89, 1.37y/ n.r. | (A)Shujinjiedu formula_1 + (B) (for 8 weeks, n=31) | (B)Levodopa 1.5g (n=31) | (1)Global improvement of symptoms | 8 weeks | (1)RR 1.15 [0.98, 1.36] | U,U,H,U,U,L |  |  |
|  |  |  |  | (2)Adverse event rate |  | (2)RR 0.36 [0.13, 1.02] |  |  |  |
| Wang 2009 b [57] | 3.5/ Webster scale 18.86, 1.74 | (A)Shujinjiedu formula_2 (1 session: 1 months, total 2 sessions, n=15) | (B)Levodopa 750mg (n=15) | (1)Global improvement of symptoms | 2 months | (1)RR 1.30 [0.86, 1.96] | U,U,H,U,U,L |  |  |
|  |  |  |  | (2)Webster scale |  | (2)MD 0.69 [-0.39, 1.77] |  |  |  |
| Shen 2008[58] | <6 months/ H-Y staging 1 to 2.5 | (A)Tongxinluo capsule plus Liuweidihuang wan + (B) (for 6 months, n=30) | (B)Madopar ( n=30) | (1)UPDRSIIIsubscore | 6 months | (1)MD -4.93 [-9.59, -0.27] | U,U,H,U,U,L |  |  |
| Liang 2008[59] | 10.9, 3.93 y/ H-Y staging 2.4, 0.88 | (A)Wuhuzhuifeng san + madopar 375 to 1000mg (for 3 months, n=30) | (B)Madopar 375 to 1000mg + trastal 100mg (n=30) | (1)Global improvement of symptoms | 3 months | (1)RR 1.24 [0.94, 1.63] | U,U,H,U,U,U |  |  |
|  |  |  |  | (2)UPDRS II subscore |  | (2)MD -3.26 [-6.46, -0.06] |  |  |  |
|  |  |  |  | (3)UPDRSIIIsubscore |  | (3)MD -3.74 [-7.63, 0.15] |  |  |  |
| Li 2008[60] | 3.55, 1.63 y/ n.r. | (A)Xifengdingzhan tang + (B) (for 3 months, n=27) | (B)Madopar (n=27) | (1)Global improvement of symptoms | 3 months | (1)RR 1.32 [1.01, 1.72] | U,U,H,U,U,L |  |  |
|  |  |  |  | (2)Dose of madopar |  | (2)MD -187.01 [-247.25, -126.77] |  |  |  |
| Ma 2005[61] | n.r./ Webster scale 14, 5.46 | (A)Xifengdingzhan wan + (B) (for 3 months, n=30) | (B)Madopar 750mg (n=30) | (1)Global improvement of symptoms | 12 weeks | (1)RR 1.47 [1.20, 1.81] | U,U,H,U,U,L |  |  |
|  |  |  |  | (2)Total UPDRS score |  | (2)MD -5.41 [-6.96, -3.86] |  |  |  |
|  |  |  |  | (3)Adverse event rate |  | (3)RR 0.41 [0.21, 0.80] |  |  |  |
| Ma 2008 a [62] | 17.6, 5.87 m/ n.r. | (A)Xifengdingzhan wan + (B) (for 1 months, n=30) | (B)Madopar 250 to 1000 mg (n=30) | (1)Global improvement of symptoms | 1 months | (1)RR 1.47 [1.20, 1.81] | U,U,H,U,U,L |  |  |
| Ma 2008 b [63] | 5.84, 1.21 y/ total UPDRS score 20.88, 3.07 | (A)Xifengdingzhan wan + madopar 250 to 750mg (for 12 weeks, n=40) | (B)Placebo+ madopar 250 to 750mg (n=40) | (1)Global improvement of symptoms | 12 weeks | (1)RR 1.44 [1.11, 1.87] | U,U,L,L,U,L |  |  |
|  |  |  |  | (2)Total UPDRS score |  | (2)MD -5.10 [-6.57, -3.63] |  |  |  |
| Cheng 2007[64] | n.r./ total UPDRS score 50.53, 6.13 | (A)Xifengdingzhan wan + (B) (for 12 weeks, n=20) | (B)Madopar (n=20) | (1)Total UPDRS score | 12 weeks | (1)MD -5.41 [-6.96, -3.86] | U,U,H,U,U,L |  |  |
|  |  |  |  | (2)Dose of madopar |  | (2)MD -165.63 [-304.10, -27.16] |  |  |  |
|  |  |  |  | (3)Adverse event rate |  | (3)RR 0.41 [0.21, 0.80] |  |  |  |
| Lu 2009[65] | 3.94, 2.39 y/ Total UPDRS score 41.06, 8.09 | (A)Xifengzhizhan tang + (B) (for 3 months, n=31) | (B)Madopar 250 to 2000mg (n=31) | (1)Global improvement of symptoms | 3 months | (1)RR 1.50 [1.08, 2.08] | U,U,H,L,U,L |  |  |
|  |  |  |  | (2)Total UPDRS score |  | (2)MD -8.89 [-13.29, -4.49] |  |  |  |
|  |  |  |  | (3)Dose of madopar |  | (3)MD -185.24 [-245.56, -124.92] |  |  |  |
| Wang 2008 a [66] | n.r./ Webster scale 17.92, 0.94 | (A)Yangganxifeng recipe + (B) (for 6 weeks, n=21) | (B)Madopar 375 to 500mg (n=21) | (1)Global improvement of symptoms | 6 weeks | (1)RR 1.20 [0.87, 1.66] | U,U,H,U,U,U |  |  |
|  |  |  |  | (2)Webster scale |  | (2)MD -3.00 [-4.14, -1.86] |  |  |  |
| Li 2009[67] | 4.5, 4.27/ n.r. | (A)Yiguan jian plus Dabuyin wan + (B) (for 3 months, n=20) | (B)Madopar (n=20) | (1)Global improvement of symptoms | 3 months | (1)RR 1.36 [1.00, 1.84] | U,U,H,U,U,L |  |  |
|  |  |  |  | (2)Dose of madopar |  | (2)MD -187.01 [-257.00, -117.02] |  |  |  |
| Chen 2009[68] | 5.18, 2.59/ .r. | (A)Yiyuan yin + (B) (for 3 months, n=46) | (B)Madopar (n=25) | (1)NMSQuest-T value | 3 months | (1)MD -1.00 [-3.19, 1.19] | U,U,L,U,U,L |  |  |
|  |  |  |  | (2)Dose of Madopar |  | (2)MD -65.00 [-144.51, 14.51] |  |  |  |
|  |  |  |  | (3)Constipation (NMP) |  | (3)RR 0.54 [0.29, 1.03] |  |  |  |
| Yang 2002[69] | n.r./ n.r. | (A)Yizhan tang + (B) (for 12 weeks, n=40) | (B)Madopar 500mg to 1200mg, bromocriptine 2.5mg (n=32) | (1)Global improvement of symptoms | 12 weeks | (1)RR 1.27 [0.98, 1.65] | U,U,H,U,U,L |  |  |
|  |  |  |  | (2)Dose of madopar |  | (2)MD -120.00 [-148.48, -91.52] |  |  |  |
|  |  |  |  | (3)Doseofbromocriptine |  | (3)MD -2.50 [-2.75, -2.25] |  |  |  |
| Pan 2009[70] | 5.54, 3.53 y/ H-Y staging 1 to 4 | (A)Zengxiao Anshen Zhichan 2 recipe + western drug (1 session: 3 weeks, total 3 session, n=62) | (B)Placebo + western drug (n=34) | (1)Total UPDRS score | 13 weeks | (1)MD -3.20 [-11.34, 4.94] | L,U,L,L,U,L |  |  |
|  |  |  |  | (2)UPDRS I subscore |  | (2)MD -0.20 [-0.83, 0.43] |  |  |  |
|  |  |  |  | (3)UPDRS II subscore |  | (3)MD -0.60 [-5.11, 3.91] |  |  |  |
|  |  |  |  | (4)UPDRS III subscore |  | (4)MD -2.90 [-8.53, 2.73] |  |  |  |
|  |  |  |  | (5)UPDRS IV subscore |  | (5)MD -0.20 [-0.95, 0.55] |  |  |  |
| Feng 2002[71] | 3.75, 0.34 y/ n.r. | (A)ZhenChan shu (for 3 months, n=30) | (B)Madopar (n=20) | (1)Global improvement of symptoms | 3 months | (1)RR 1.08 [0.84, 1.41] | U,U,U,U,U,L |  |  |
| Wu 2008 b [72] | 11.5, 6.3 y/ n.r. | (A)Zhenchan tang + (B) (for 8 weeks, n=30) | (B)Madopar 187.5 to 1125 mg (n=25) | (1)Global improvement of symptoms | 8 weeks | (1)RR 1.11 [0.91, 1.35] | U,U,H,U,U,L |  |  |
| Wang 2008 b [73] | 4.3 y/ total UPDRS score 103.58, 1.95 | (A)Zhichanshudu tang (for 30 days, n=20) | (B)Levodopa 750mg (n=15) | (1)Global improvement of symptoms | 30 days | (1)RR 1.20 [0.79, 1.83] | U,U,H,U,U,L |  |  |
|  |  |  |  | (2)Total UPDRS score |  | (2)MD -9.16 [-10.68, -7.64] |  |  |  |
|  |  |  |  | (3)Webster scale |  | (3)MD 0.39 [-0.73, 1.51] |  |  |  |
| Wang 2010[74] | >6 m/ H-Y staging 2.1, 0.48 | (A)Zhizhan tang + (B) (twice per day for 12 weeks, n=30) | (B)Madopar 500mg (n=30) | (1)Global improvement of symptoms | 12 weeks | (1)RR 1.00 [0.94, 1.07] | L,U,H,U,U,L |  |  |
|  |  |  |  | (2)Total UPDRS score |  | (2)MD -1.30 [-2.36, -0.24] |  |  |  |
|  |  |  |  | (3)UPDRS I subscore |  | (3)MD 0.10 [-0.18, 0.38] |  |  |  |
|  |  |  |  | (4)UPDRS II subscore |  | (4)MD -0.60 [-1.26, 0.06] |  |  |  |
|  |  |  |  | (5)UPDRS III subscore |  | (5)MD -0.63 [-1.32, 0.06] |  |  |  |
|  |  |  |  | (6)UPDRS IV subscore |  | (6)MD -0.17 [-0.54, 0.20] |  |  |  |
| Shen 2006[75] | 4.13, 2.17 y/ H-Y staging 2.70, 0.59,  Webster scale 17.76, 6.63 | (A)Zibuganshen recipe + (B) (for 3 months, n=40) | (B)Madopar, sinemet, artane (n=32) | (1)Global improvement of symptoms | 3 months | (1)RR 1.00 [0.95, 1.06] | U,U,H,L,U,L |  |  |
|  |  |  |  | (2)Total UPDRS score |  | (2)MD -10.74 [-20.74, -0.74] |  |  |  |
|  |  |  |  | (3)Webster scale |  | (3)MD -2.86 [-5.68, -0.04] |  |  |  |
|  |  |  |  | (4)Nausea & vomiting (NMP) |  | (4)RR 0.36 [0.14, 0.94] |  |  |  |
|  |  |  |  | (5)Constipation (NMP) |  | (5)RR 0.49 [0.23, 1.04] |  |  |  |
| Jiang 2003[76] | A:5 y, B:4 y/ n.r. | (A)Zibuganshen Huoxiexifeng recipe + (B) (for 30 days, n=15) | (B)Madopar 375mg tid, artane 6mg (n=12) | (1)Global improvement of symptoms | 1 year | (1)RR 1.12 [0.84, 1.49] | U,U,H,U,U,U |  |  |
| Tang 2005[77] | n.r./ Webster scale 11.9, 1.85 | (A)Ziyinxifeng granule (for 2 months, n=30) | (B)Madopar 250 to 500mg (n=30) | (1)Global improvement of symptoms | 2 months | (1)RR 0.96 [0.76, 1.22] | U,U,U,U,U,L |  |  |
|  |  |  |  | (2)Webster scale |  | (2)MD -0.50 [-0.90, -0.10] |  |  |  |
| Wang 2004[78] | 6 m to 3 y/ H-Y staging 1 to 3 | (A)Ziyinxifenghuoxie tang + Madopar 250 to 500mg (1 session: 1 week, total 3 sessions, n=20) | (B)Placebo+Madopar 250 to 500mg (n=20) | (1)Global improvement of symptoms | 3 months | (1)RR 1.07 [0.76, 1.49] | L,L,L,U,U,L |  |  |
|  |  |  |  | (2)Total UPDRS score |  | (2)MD -3.40 [-8.46, 1.66] |  |  |  |
| Cui 2004[79] | n.r./ n.r. | (A)Herbal recipe_no name + (B) (n.r. , n=50) | (B)Madopar (n=50) | (1)Global improvement of symptoms | 1 year | (1)RR 2.88 [1.90, 4.34] | U,U,H,U,U,L |  |  |
| Qiu 1998[80] | n.r./ n.r. | (A)Individualized herbal recipe + (B) (for 6 months, n=35) | (B)Madopar 750mg , amatadine 300mmg tid, artane 6mg (n=20) | (1)Global improvement of symptoms | 6 months | (1)RR 1.27 [0.89, 1.82] | U,U,H,U,U,L |  |  |
| Ma 2003[81] | A: 4 y, B:3.5 y/ n.r. | (A)Individualized herbal recipe + (B) (for 3months, n=42) | (B)Levodopa 0.5 to 4g, artane 6.5 to 15mg (n=30) | (1)Global improvement of symptoms | 3 months | (1)RR 1.13 [0.92, 1.39] | U,U,U,U,U,L |  |  |
| Wang 2005 b [82] | n.r./ U PDRS II+III subscore 32.91, 13.33 | (A)Individualized herbal recipe + (B) (1 session: 30 days, total 3 sessions, n=53) | (B)Madopar <750mg, amantadine 100 to 200mg (n=50) | (1)Global improvement of symptoms | 3 months | (1)RR 1.16 [0.98, 1.38] | U,U,H,U,U,L |  |  |
|  |  |  |  | (2)UPDRS II + III Subscore |  | (2)MD -6.97 [-11.65, -2.29] |  |  |  |

RR: risk ratio; MD: mean difference; UPDRS I subscore: mentation, behavior and mood; UPDRS II subscore: activities of daily living; UPDRS III subscore: motor function; UPDRS IV subscore: complications of therapy; H-Y staging: Hoehn and Yahr Staging; HAM-D: Hamilton depression rating scale; PDQ-39: 39 item Parkinson’s disease questionnaire; Dose of madopar and levodopa (mg/d); NMP: non-movement problems: NMSQuest: total non-motor symptoms score value; risk of bias assessment: random sequence generation, allocation concealment, blinding of participants, personnel or outcome assessors, incomplete outcome data, selective reporting and other bias; L: low risk of bias; H: high risk of bias; U: unclear; n.r.: not reported
